# Composition of Surface Layer at the Water–Air Interface and Micelles of Triton X-100 + Rhamnolipid Mixtures

**DOI:** 10.1007/s10953-017-0642-7

**Published:** 2017-06-15

**Authors:** Diana Mańko, Anna Zdziennicka, Bronisław Jańczuk

**Affiliations:** Department of Interfacial Phenomena, Faculty of Chemistry, Maria Curie-Skłodowska University, Maria Curie-Skłodowska Sq. 3, 20-031 Lublin, Poland

**Keywords:** Adsorption, Aggregation, Micellization, Surfactants, Rhamnolipid, Triton X-100

## Abstract

Measurements of the surface tensions, densities and viscosities of aqueous solutions of Triton X-100 (TX-100) and rhamnolipid (RL) mixtures, at constant concentration of RL or TX-100, were carried out. The measured values of the surface tension were compared to those determined using different theoretical models and on the basis of the surface tension of aqueous solutions of individual surfactants. From the surface tension isotherms, the Gibbs surface excess concentration of TX-100 and RL, the composition of surface layer and the standard Gibbs free energy of adsorption at the water–air interface were determined. Moreover, on the basis of surface tension, density and viscosity isotherms, the CMC of surfactants mixtures were evaluated. From the density isotherms, apparent and partial molar volumes of TX-100 and RL were also determined. These volumes were compared to those calculated from the sizes of TX-100 and RL molecules. There was observed a synergetic effect in the reduction of water surface tension and micelle formation, which was confirmed by the intermolecular interactions parameter. In the case of micelle formation, this effect was discussed based on the standard Gibbs free energy of micellization as well as of TX-100 and RL mixing in the micelles. The synergism of TX-100 and RL mixtures in the reduction of water surface tension and micelle formation was explained on the basis of electrostatic interactions between the hydrophilic part of TX-100 and RL molecules; this was supported by pH measurements.

**Electronic supplementary material:**

The online version of this article (doi:10.1007/s10953-017-0642-7) contains supplementary material, which is available to authorized users.

## Introduction

Recently, increasing numbers of investigations aimed at reducing of use of synthetic surfactants, particularly non-biodegradable and toxic ones, and substituting them with new more environmentally and human friendly ones have been reported. Of specific interest are biocompatible surfactants including biosurfactants. Biosurfactants are characterized by high surface activity, specificity of action, temperature stability and resistance to pH changes. They undergo biodegradation more readily and are less toxic [1–3]. Owing to such properties, biosurfactants are applied in the cosmetic, agricultural, textile, cellulose and stationary industries, among others [4–6]. They are also exploited in crude oil recovery and in the remediation and detoxification of soil [7]. Biosurfactants are also characterized by antimicrobial properties owing to which they have been used as antibiotics for many years [8, 9]. Moreover, they change the interfacial tension so they can protect various kinds of implant surfaces such as urological catheters against the adhesion of micro-organisms and, as a result, they protect patients against complications such as infectious diseases [10]. However, the application of biosurfactants is limited by the high cost of their production [11]. From the economic point of view it seems that the use of such surfactants as additives to synthetic ones is more suitable. Mixtures of biosurfactants and classical surfactants can exhibit a synergetic effect in the reduction of water surface tension and micelle formation [12, 13]. It seems that one of interesting mixtures may be those of Triton X-100 and rhamnolipid. TX-100 is a typical non-ionic surfactants belonging to the alkyl polyethylene oxide family. It is used to solubilize membrane phospholipids, in DNA extraction and in the purification of membrane-bound proteins and enzymes, without a loss of their biological activity [14–16], and in almost every type of liquid, paste, and powdered cleaning compound, ranging from heavy-duty industrial and agrochemical products to gentle detergents [17]. Unfortunately, the literature lacks studies dealing with the adsorption and aggregation properties of these surfactant mixtures. Therefore, the aim of our studies was to determine the surface and volumetric properties of mixtures of Triton X-100 and rhamnolipid, using measurements of the surface tension, density and viscosity of the aqueous solutions of Triton X-100 (TX-100) and rhamnolipid (RL) mixtures at the constant concentration of RL or TX-100.

## Utility Equations for the Data Treatment

The surface tensions of the aqueous solutions of RL and TX-100 were calculated by several methods and these were compared to the measured values. In these calculations the equations of independent adsorption, Joos, Szyszkowski and Fainerman and Miller were applied.

If independent adsorption takes place, the following equation is satisfied:

1 $$ \gamma_{\text{LV}} = \gamma_{\text{W}} - \pi_{1} - \pi_{2} $$

where *γ*_LV_ is the surface tension of the aqueous solution of RL and TX-100 mixture, *γ*_W_ is the water surface tension, *π*_1_ and *π*_2_ are the differences between the surface tensions of water and aqueous solution of TX-100 and RL [18, 19], respectively.

The Joos equation [20] for the mixture of TX-100 and RL in which TX-100 represents a non-ionic surfactant and RL an ionic being a type of 1:1 electrolyte (A^+^ and B^−^) can be written in the form [21]:

2 $$ \exp \left( {\frac{ - \pi }{{RT\Gamma_{0}^{\infty } }}} \right) + \exp \left( {\frac{ - \pi }{{RT\Gamma_{1}^{\infty } }}} \right)\frac{{C_{1} }}{{a_{1} }} + \exp \left( {\frac{ - \pi }{{2RT\Gamma_{2}^{\infty } }}} \right)\frac{{C_{2} }}{{a_{2} }} = 1 $$

if their activities are close to the concentrations (*C*) (for *C* < 10^−2^ mol·dm^−3^), where $\Gamma_{0}^{\infty }$, $\Gamma_{1}^{\infty }$ and $\Gamma_{2}^{\infty }$ are the limiting adsorption of the solvent, TX-100 and RL, respectively, *π* is the difference between the surface tensions of water and the aqueous solution of TX-100 and RL, *R* is the gas constant, *T* is the absolute temperature, *C*_1_ and *C*_2_ are TX-100 and RL concentrations, respectively, and *a*_1_ and *a*_2_ are the constants characteristic of a given surfactant.

The limiting maximal surface excess concentrations of TX-100 ($\Gamma_{1}^{\infty }$) and RL ($\Gamma_{2}^{\infty }$) and the *a*_1_ and *a*_2_ values can be determined from the Joos equation for the aqueous solutions of individual surfactants [20–22] as:

3 $$ \exp \left( {\frac{{ - \pi_{1} }}{{RT\Gamma_{0}^{\infty } }}} \right) + \exp \left( {\frac{{ - \pi_{1} }}{{RT\Gamma_{1}^{\infty } }}} \right)\frac{{C_{1} }}{{a_{1} }} = 1 $$

and

4 $$ \exp \left( {\frac{{ - \pi_{2} }}{{RT\Gamma_{0}^{\infty } }}} \right) + \exp \left( {\frac{{ - \pi_{2} }}{{2RT\Gamma_{2}^{\infty } }}} \right)\frac{{C_{2} }}{{a_{2} }} = 1 $$

The Szyszkowski equation [13, 23] for the RL and TX-100 mixture can be expressed in the following form:

5 $$ \gamma_{\text{o}} - \gamma_{\text{LV}} = nRT\Gamma_{m} \ln \left( {\frac{C}{b} + 1} \right) $$

where *γ*_*o*_ is the solute surface tension, *n* is equal to one for TX-100 and 2 for RL, Γ_*m*_ is the maximum Gibbs surface excess concentration of surfactant at the water–air interface and *b* is a constant.

Taking into account the ideal mixing of homologous surfactants, Fainerman and Miller proposed an equation of state which can be written as [24, 25]:

6 $$ { \exp }\overline{\varPi } = { \exp }\overline{\varPi }_{1} + { \exp }\overline{\varPi }_{2} - 1 $$

where $\overline{\varPi } = \pi \omega /RT$, $\overline{\varPi }_{1} = \pi {}_{1}\omega_{1} /RT$, $\overline{\varPi }_{2} = \pi_{2} \omega_{2} /RT$ are the dimensionless surface pressures of the mixture and individual solutions of TX-100 and RL, respectively, *ω*_1_, *ω*_2_ and *ω* are the molar surface areas of TX-100, RL and their mixture, respectively. In the case of TX-100 and RL *ω*_1_ and *ω*_2_ are equal to 2.15 × 10^5^ and 4.16 × 10^5^ m^2^·mol^−1^, respectively. The surface pressures of TX-100 and RL were taken from the literature [18, 19].

For determination of the Gibbs surface excess concentration of TX-100 (Γ_1_) and RL (Γ_2_), the equation of the Gibbs adsorption isotherm was used in the following form [13, 22]:

7 $$ \Gamma_{i} = - \frac{{C_{i} }}{nRT}\left( {\frac{{d\gamma_{\text{LV}} }}{{d\ln C_{i} }}} \right)_{{T,C_{j \ne i} }} = - \frac{1}{2.303nRT}\left( {\frac{{d\gamma_{\text{LV}} }}{{d\log_{10} C_{i} }}} \right)_{{T,C_{j \ne i} }} $$

where *C*_*i*_ is the concentration of component *i*, *j* is the number of components.

Knowing the Gibbs surface excess concentration of TX-100 and RL it is possible to determine the composition of the surface layer based on the Chattoraj and Birdi equation [26]:

8 $$ \Gamma_{\text{o}}^{{}} NA_{0}^{w} + \Gamma_{1}^{{}} NA_{1} + \Gamma_{2}^{{}} NA_{2} = 1 $$

where *N* is the Avogadro number, $A_{0}^{w}$, *A*_1_ and *A*_2_ are the excluded areas of water (10 Å^2^), TX-100 (35.7 Å^2^) and RL (69.08 Å^2^), respectively (the excluded area is the area of the interface unavailable to one molecule due to the presence of another), and Γ_o_ is the Gibbs surface excess concentration of water.

From Eq. 8 the number of water moles in 1 m^2^ can be calculated if the Gibbs surface excess concentrations of TX-100 and RL are known. Then the surface mole fractions of $X_{1}^{0}$ and $X_{2}^{0}$ forming a 1 m^2^ surface plane can be estimated from the following equations:

9 $$ X_{1}^{0} = \frac{{\Gamma_{1}^{{}} }}{{\Gamma_{0}^{{}} + \Gamma_{1}^{{}} + \Gamma_{2}^{{}} }} $$

10 $$ X_{2}^{0} = \frac{{\Gamma_{2}^{{}} }}{{\Gamma_{o}^{{}} + \Gamma_{1}^{{}} + \Gamma_{2}^{{}} }} $$

Knowing Γ_1_ and Γ_2_ it is also possible to determine the relative mole fraction of TX-100 ($X_{1}^{\text{S}}$) and RL ($X_{2}^{\text{S}}$) in the surface layer at the solution–air interface from the following expressions:

11 $$ X_{1}^{\text{S}} = \frac{{\Gamma_{1} }}{{\Gamma_{1} + \Gamma_{2} }} $$

12 $$ X_{2}^{\text{S}} = 1 - X_{1}^{\text{S}} $$

The mole fractions of TX-100 and RL in the surface monolayer can be also determined on the basis of the Hua, Rubingh and Rosen theory [13, 27, 28]. They derived the following equation for calculation of the surface mixed monolayer composition:

13 $$ \frac{{\left( {X_{{_{1} }}^{\text{S}} } \right)^{2} \ln \left( {\alpha C_{12} /X_{{_{1} }}^{\text{S}} C_{1}^{0} } \right)}}{{\left( {1 - X_{{_{1} }}^{\text{S}} } \right)^{2} \ln \left[ {\left( {1 - \alpha } \right)C_{12} /\left( {1 - X_{{_{1} }}^{\text{S}} } \right)C_{2}^{0} } \right]}} = 1 $$

where *α* is the mole fraction of RL in the bulk phase, (1 − *α*) is the mole fraction of TX-100 in the bulk phase, $C_{1}^{0}$, $C_{2}^{0}$ and *C*_12_ are the molar concentrations of TX-100, RL and their mixture in the bulk phase, respectively, required to produce a given surface tension value.

Knowing the mole fractions of the components in the mixed monolayer, the parameter of the intermolecular interactions of surfactant molecules in this layer (*β* ^*δ*^) was calculated from the Hua, Rubingh and Rosen equation, which has the form [13, 27, 28]:

14 $$ \beta^{\delta } = \frac{{\ln (\alpha C_{12} /X_{{_{1} }}^{\text{S}} C_{1}^{0} )}}{{(1 - X_{{_{1} }}^{\text{S}} )^{2} }} $$

The value which determines adsorption of a given surfactant at the water–air interface is the Gibbs standard free energy of adsorption ($\Delta G_{{_{\text{ads}} }}^{\text{o}}$). This energy can be determined using different methods, among which the Langmuir equation modified by de Boer is commonly used [29]:

15 $$ \frac{{A_{0} }}{{A - A_{0} }}\exp \frac{{A_{0} }}{{A - A_{0} }} = \frac{C}{\varpi }\exp \left( {\frac{{ - \Delta G_{\text{ads}}^{ 0} }}{RT}} \right) $$

where *A* is the area occupied per molecule of a given surfactant at the water–air interface, *A*_0_ is the excluded area of this surfactant and *ϖ* is the number of water moles in 1 dm^3^, equal to 55.41 at 293 K.

If the changes of surface tension as a function of surfactant concentration can be described by the Szyszkowski equation [13, 22], then the constant *b* in this equation at 293 K satisfies the requirement:

16 $$ b = \varpi { \exp }\left(\frac{{\Delta G_{\text{ads}}^{\text{o}} }}{RT}\right)$$

Based on the isotherms of surface tension, density and viscosity, the critical micelle concentration of the TX-100 and RL mixtures were determined. The results were compared to those calculated for the ideal mixture of surfactants from the equation [13]:

17 $$ \frac{1}{{CMC_{12} }} = \frac{\alpha }{{CMC_{1} }} + \frac{1 - \alpha }{{CMC_{2} }} $$

where *CMC*_1_, *CMC*_2_ and *CMC*_12_ are the critical micelle concentrations of TX-100, RL and their mixtures, respectively.

The volume of surfactant molecules can be changed during the transfer from the bulk phase to the micelle; therefore there were calculated using the apparent (*φ*_*V*_) and partial ($\overline{V}_{\text{m}}$) molar volumes of TX-100 and RL from the Kale and Zana [30] and Benjamin [31] equations:

18 $$ \varphi_{V} = \frac{{M_{\text{S}} }}{{\rho_{0} }} + \frac{{1000\left( {\rho_{0} - \rho } \right)}}{{C_{S} \rho_{0} }} $$

19 $$ \overline{V}_{\text{m}} = \frac{{M_{\text{S}} }}{\rho }\left[ {1 - \frac{{\left( {100 - C_{p} } \right)}}{\rho }\frac{d\rho }{{dC_{\% } }}} \right] $$

where *M*_S_ is the molecular weight of surfactant, *C*% is the percentage weight of the solute, *C*_S_ is the concentration of surfactant in mol·cm^−3^, *ρ* is the density of the solution, and *ρ*_0_ is the density of the aqueous solution of TX-100 or RL.

To determine $\overline{V}_{\text{m}}$, it is necessary to know the dependence between the density and *C*%. For all studied solutions at the constant concentration of one surfactant (*C*_1_ or *C*_2_), this dependence can be fitted by the following polynomial:

20 $$ \rho = c + kC_{\% } + sC_{\% }^{2} $$

where *c*, *k* and *s* are constants.

Apparent and partial molar volumes should depend on the mole fractions of TX-100 and RL in the micelles. Therefore, they were determined from the Hua, Rubingh and Rosen equation [13, 27, 28]:

21 $$ \frac{{\left( {X_{1}^{\text{M}} } \right)^{2} \ln \left( {\alpha CMC_{12}^{{}} /X_{1} CMC_{1}^{{}} } \right)}}{{\left( {1 - X_{1}^{\text{M}} } \right)^{2} \ln \left[ {\left( {1 - \alpha } \right)CMC_{12}^{{}} /\left( {1 - X_{1}^{\text{M}} } \right)CMC_{2}^{{}} } \right]}} = 1 $$

where $X_{1}^{\text{M}}$ is the mole fraction of TX-100 in the mixed micelle.

Knowing the mole fraction of TX-100 and RL in the mixed micelle, the parameter of intermolecular interactions (*β* ^M^) was calculated [13, 27, 28]:

22 $$ \beta^{\text{M}} = \frac{{\ln (\alpha CMC_{12}^{{}} /X_{1}^{\text{M}} CMC_{1}^{{}} )}}{{(1 - X_{1}^{\text{M}} )^{2} }} $$

The standard Gibbs free energy of micellization ($\Delta G_{\text{mic}}^{\text{o}}$) was calculated from the following equation [32]:

23 $$ \Delta G_{\text{mic}}^{\text{o}} = \Delta G_{\text{mic}}^{\text{mid}} + \Delta G_{\text{mic}}^{\text{E}} = \left[ {\left( {1 - \alpha } \right)\Delta G_{\text{mic(1)}}^{\text{o}} + \alpha \Delta G_{\text{mic(2)}}^{\text{o}} } \right] + \left[ {RT\left( {X_{1}^{\text{M}} \ln f_{1} + X_{2}^{\text{M}} \ln f_{2} } \right)} \right] $$

where $\Delta G_{\text{mic}}^{\text{mid}}$ is the standard Gibbs free energy of ideal mixture micellization, $\Delta G_{\text{mic}}^{\text{E}}$ is the excess Gibbs free energy of non-ideal mixing of surfactants in the micelle, and *f*_1_ and *f*_2_ are the activity coefficients of TX-100 and RL in the mixed micelles, which can be calculated from the following equations [13]:

24 $$ \ln f_{1} = \beta^{\text{M}} \left( {1 - X_{1}^{\text{M}} } \right)^{2} $$

and

25 $$ \ln f_{2} = \beta^{\text{M}} \left( {X_{1}^{\text{M}} } \right)^{2} . $$

## Experimental

### Chemicals

**Fig. 1:**
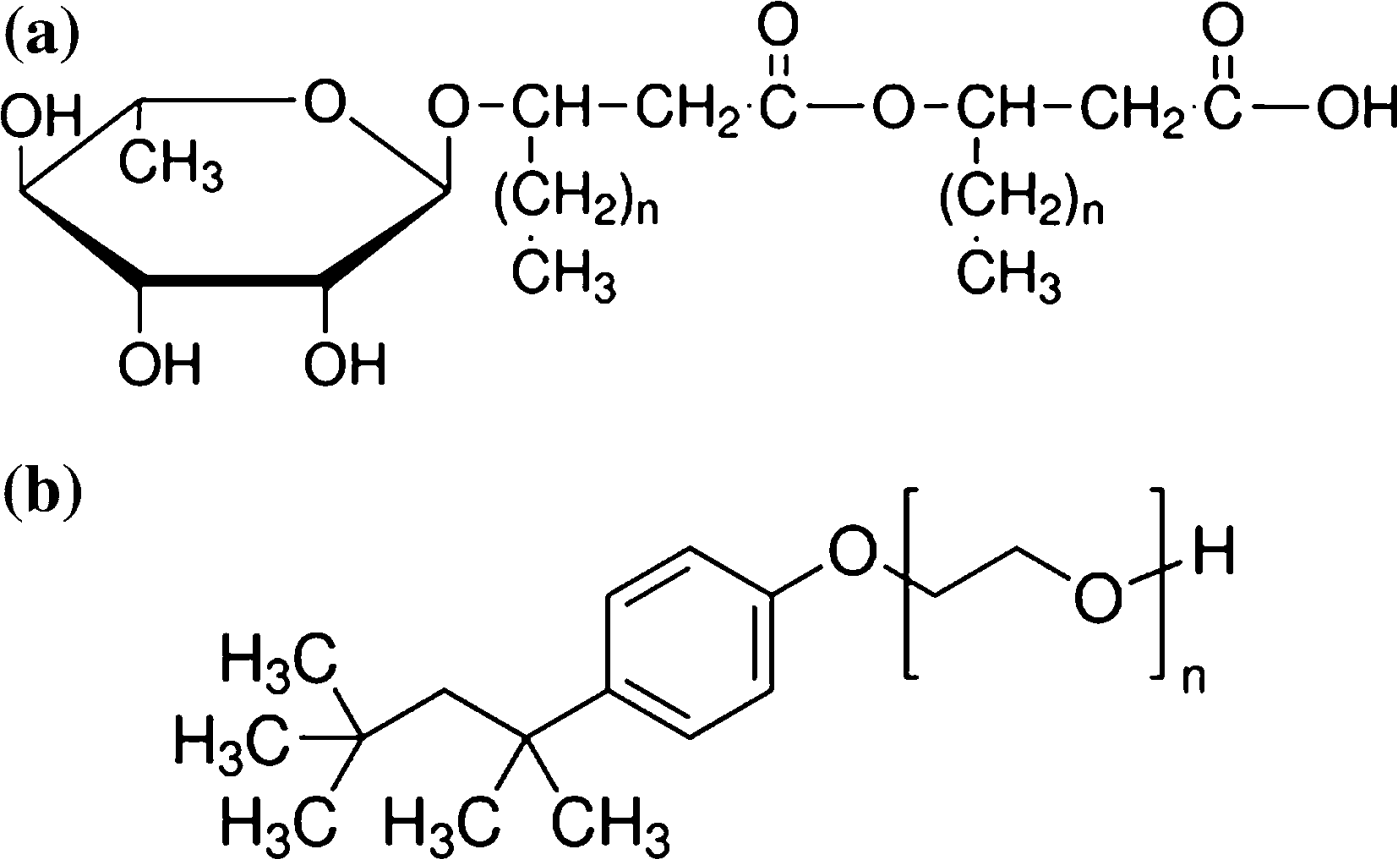
Structures of RL and TX-100 molecules

In our studies R-95 Rhamnolipid (RL) (Fig. 1a), *p*-(1,1,3,3-tetramethylbutyl) phenoxypoly(ethylene glycol) (Triton X-100 or TX-100) (Fig. 1b), and doubly distilled and deionized water (Destamat Bi18E) (internal specific resistance equal to 18.2 MΩ) were used. RL and TX-100 were purchased from Sigma–Aldrich.

Rhamnolipids are produced by *Pseudomonas aeruginosa* and grow on various carbon sources. That can be a mixture of different homologues, among which the mono- (with an average molecular weight of 504) and dirhamnolipids (with an average molecular weight of 650) are present in large amounts. In our studies R-95 Rhamolipid containing 95% of the monorhamnolipid form was used. The “Triton X” series of detergents are produced from octylphenol polymerized with ethylene oxide. The number (“−100”) relates only indirectly to the number of ethylene oxide units in the structure. TX-100 has an “average of 9.5” ethylene oxide units per molecule, with an average molecular weight of 625. In our studies Triton X-100 BioExtra containing less than 0.5% of impurities was used. Among the impurities, phosphorus (≤0.05%), sodium (≤0.1%), ammonium (≤0.05%), chloride (≤0.05%) and sulfate (≤0.05%) are found in large amounts. Both surfactants were used without further purification.

### Solution Preparation

Two series of the aqueous solutions of RL and TX-100 mixtures were prepared using doubly distilled and deionized water. The first one included solutions at the constant TX-100 concentrations (*C*_1_) equal to 1 × 10^−8^, 5 × 10^−8^, 1 × 10^−7^, 5 × 10^−7^, 1 × 10^−6^, 5 × 10^−6^, 1 × 10^−5^, 5 × 10^−5^, 1 × 10^−4^, 2 × 10^−4^, 4 × 10^−4^, 6 × 10^−4^, 8 × 10^−4^, 1 × 10^−3^, 2 × 10^−3^ mol·dm^−3^. In each series of solutions, at a given constant concentration of TX-100, the concentration of RL was changed in the range from 0.0002 to 40 mg·dm^−3^. The second series of solutions was prepared at the constant RL concentrations (*C*_2_) equal to 0.0002, 0.0005, 0.00125, 0.003, 0.00625, 0.01, 0.02, 0.05, 0.125, 0.5, 1, 5, 10, 20, 32 and 40 mg·dm^−3^. In each series of solutions at a given, constant concentration of RL, the concentration of TX-100 was changed in the range from 1 × 10^−8^ to 2 × 10^−3^ mol·dm^−3^.

### Measurements

The equilibrium surface tension (*γ*_LV_) of the aqueous solutions of RL and TX-100 mixtures was measured with a Krüss K100C tensiometer using both the platinum ring detachment and the Wilhelmy plate methods. The ring and plate were cleaned with distilled water and heated to red color before each measurement. In all cases 10 or more successive measurements were performed. The root-mean-square deviation of the surface tension data depending on the surfactant concentration range is from ±0.1 to ±0.2 mN·m^−1^ and the standard uncertainty (standard deviation of the mean) is in the range from ±0.025 mN·m^−1^ (from 16 values for each surfactants mixture concentration in the range of low concentration) to ±0.063 mN·m^−1^ (from 10 values for each surfactants mixture concentrations in the range of high concentration), respectively.

A DMA 5000 Anton Paar densitometer was used to measure the densities of the aqueous solutions of RL and TX-100 and their mixtures. The precision of the density measurements given by the manufacturer is ±1 × 10^−6^ g·m^−3^.

The accuracy of the density measurements and the temperature given by the manufacturer are ±1 × 10^−6^ g·m^−3^ and ±0.001 K, respectively. The relative uncertainty was calculated to be equal to ±0.01%.

Dynamic viscosity measurements of the aqueous solutions of RL and TX-100 mixtures were performed with an Anton Paar viscosimeter (AMVn) with the precision of 0.0001 mPa·s. The relative uncertainty was estimated to be ±0.3%, whereas the reproducibility does not exceed ±0.1%. The densitometer and viscosimeter were regularly calibrated with distilled and deionized water and methanol.

The pH of all solutions was natural but measured using a pH meter from Hanna Instruments (HI3220).

All the experiments were performed at 293 K within ±0.1 K.

## Results and Discussion

### Surface Tension Isotherms

The data obtained from the surface tension measurements of aqueous solutions of TX-100 and RL mixtures were considered based on four characteristic cases (Table 1). The first one deals with the mixtures in which the constant value of *C*_1_ is in the range of $C_{1}^{0}$ corresponding to formation of the TX-100 unsaturated monolayer ($C_{1}^{0}$(unsat.)) at the water–air interface [19] and *C*_2_ changes from zero to the maximum value used in the studies. The second one includes the mixtures in which *C*_1_ is in the range of $C_{1}^{0}$ corresponding to the saturated monolayer of TX-100 ($C_{1}^{0}$(sat.)) and *C*_2_ changes as in the first case. The third case deals with constant *C*_2_ which is in the range of $C_{2}^{0}$ corresponding to the unsaturated monolayer of RL at the water–air interface ($C_{2}^{0}$(unsat.)) [18] and *C*_1_ changes from zero to the maximum value used. The fourth refers to the solution in which *C*_2_ is in the range of $C_{2}^{0}$ corresponding to the saturated monolayer of RL ($C_{2}^{0}$(sat.)) and *C*_1_ changes as in the third case (Table 1). It should be pointed out that there is a characteristic point on the surface tension isotherms. This point refers to the concentrations of both surfactants corresponding to those at which their individual saturated monolayers at the water–air interface start to form [18, 19].

**Table 1:** Four cases taking into consideration for the TX-100 and RL mixtures

Number of case	*C*_1_ [mol·dm^−3^]	*C*_2_ [mol·dm^−3^]	Γ_12_[×10^−6^ mol·m^−2^]
I	*C*_1_ = $C_{1}^{0}$(unsat.)	*C*_2_ = $C_{2}^{0}$	Γ_12_ = $\Gamma_{1}^{0}$ + $\Gamma_{2}^{0}$
II	*C*_1_ = $C_{1}^{0}$(sat.)	*C*_2_ = $C_{2}^{0}$(unsat.)	Γ_12_ = $\Gamma_{1}^{0}$ + $\Gamma_{2}^{0}$
		*C*_2_ = $C_{2}^{0}$(sat.)	Γ_12_ < $\Gamma_{1}^{0}$ + $\Gamma_{2}^{0}$
III	*C*_1_ = $C_{1}^{0}$	*C*_2_ = $C_{2}^{0}$(unsat.)	Γ_12_ = $\Gamma_{1}^{0}$ + $\Gamma_{2}^{0}$
IV	*C*_1_ = $C_{1}^{0}$(unsat.)	*C*_2_ = $C_{2}^{0}$(sat.)	Γ_12_ = $\Gamma_{1}^{0}$ + $\Gamma_{2}^{0}$
	*C*_1_ = $C_{1}^{0}$(sat.)		Γ_12_ < $\Gamma_{1}^{0}$ + $\Gamma_{2}^{0}$

$C_{1}^{0}$(unsat.)—the concentration of TX-100 corresponding to its unsaturated monolayer in the absence of RL

$C_{2}^{0}$(unsat.)—the concentration of RL corresponding to its unsaturated monolayer in the absence of TX-100

$C_{1}^{0}$(sat.)—the concentration of TX-100 corresponding to its saturated monolayer in the absence of RL

$C_{1}^{0}$(sat.)—the concentration of RL corresponding to its saturated monolayer in the absence of TX-100

$\Gamma_{1}^{0}$—the Gibbs surface excess concentration of individual TX-100 in the absence of RL

$\Gamma_{2}^{0}$—the Gibbs surface excess concentration of individual RL in the absence of TX-100

**Fig. 2:**
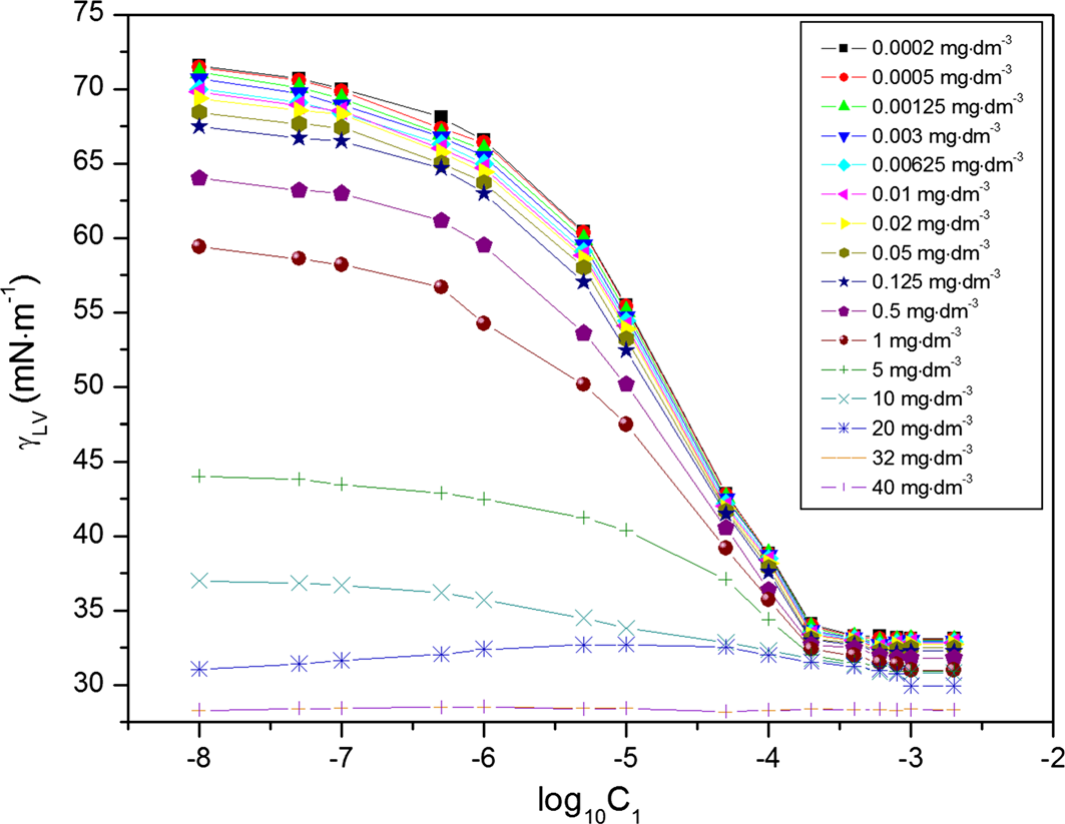
A plot of the surface tension (*γ*_LV_) of the aqueous solutions of TX-100 and RL mixtures at constant RL concentration versus log_10_ of TX-100 concentration in the bulk phase (*C*_1_). *Curves 1–16* correspond to the RL concentrations equal to 0.0002, 0.0005, 0.00125, 0.003, 0.00625, 0.01, 0.02, 0.05, 0.125, 0.5, 1, 5, 10, 20, 32 and 40 mg·dm^−3^

**Fig. 3:**
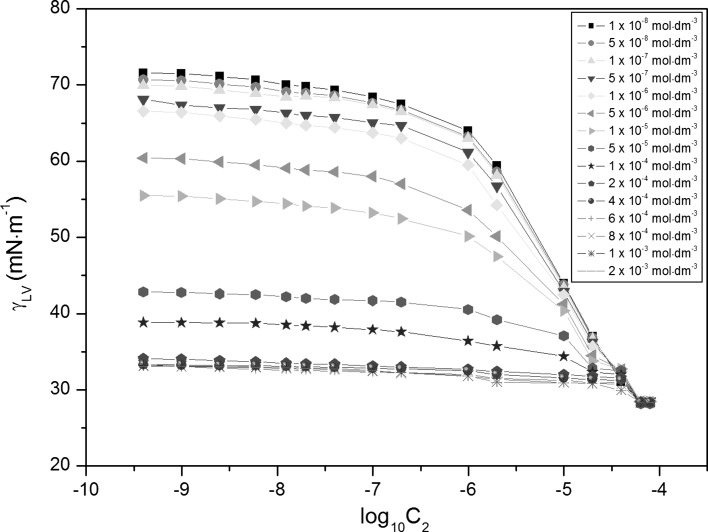
A plot of the surface tension (*γ*_LV_) of the aqueous solutions of TX-100 and RL mixtures at constant TX-100 concentration versus log_10_ of RL concentration in the bulk phase (*C*_2_). *Curves 1–15* correspond to TX-100 concentrations equal to 1 × 10^−8^, 5 × 10^−8^, 1 × 10^−7^, 5 × 10^−7^, 1 × 10^−6^, 5 × 10^−6^, 1 × 10^−5^, 5 × 10^−5^, 1 × 10^−4^, 2 × 10^−4^, 4 × 10^−4^, 6 × 10^−4^, 8 × 10^−4^, 1 × 10^−3^, 2 × 10^−3^ mol·dm^−3^

The experimental and theoretical isotherms (Eqs. 1, 2, 5, 6) [13, 18–22] of the surface tension of aqueous solutions of RL and TX-100 mixtures (Figs. 2, 3 and S1–S6), show that, in the range of *C*_1_ and *C*_2_ equal to $C_{1}^{0}$(unsat.) and $C_{2}^{0}$(unsat.) [18, 19], respectively, the surface tension of a solution is equal to the difference between the surface tension of water and the sum of the pressures of TX-100 and RL (Eq. 1) (Figs. S1–S6). In this concentration range the measured values of surface tension of the solutions are also close to those calculated from the Szyszkowski [13] (Eq. 5), Joos [20] (Eq. 2) and Fainerman and Miller [24, 25] (Eq. 6) equations. This means that under these conditions of surfactant concentration, there are negligible interactions in the mixed surface layer between the adsorbed surfactants molecules. The increase of constant concentration of one surfactant results in greater differences between the measured and theoretical values of surface tension. In the case when the constant concentration of RL is close to its individual CMC, the addition of TX-100 causes insignificant increase of solution surface tension. Knowing that the surface tension values of the aqueous solution of single TX-100 and RL at their CMC (2.9 × 10^−4^ mol·dm^−3^ [23] and 26.24 mg·dm^−3^ [18], respectively) are equal to 33.8 [23] and 27.9 mN·m^−1^ [18], we can state that the replacement of RL molecules by TX-100 in the surface layer should increase the solutions surface tension. However, this increase does not exceed the value of the surface tension of aqueous solutions of TX-100 (at CMC) equal to 33.8 mN·m^−1^. Comparing the experimental values of surface tension to those determined theoretically using different approaches, it can be stated that there is a synergetic effect in the reduction of water surface tension by the mixture of TX-100 and RL if the constant concentration of one surfactant is in the range of its concentration corresponding to the saturated monolayer at the water–air interface in the absence of the other one but lower than its CMC [18, 19].

Considering the synergetic effect of the RL and TX-100 mixtures in the reduction of water surface tension, the possibility of water surface tension increase by inorganic ions being in solution as impurities of the surfactants should be taken into account [33]. However, on one hand, the surface tension of the aqueous solutions of TX-100 and RL mixtures was compared to that of the aqueous solution of individual surfactants in the presence of the same impurities [18, 19]. On the other hand, the influence of strong electrolytes, at the concentrations corresponding to those in the solutions on the surface tension of “pure” water is practically undetectable by surface tension measurements [34]. For example, the amount of NaCl at the highest concentration of TX-100 (2 × 10^−3^ mol·dm^−3^) does not exceed 0.002 mol·kg^−1^ of water. This concentration of NaCl increases the water surface tension much less than 0.1 mN·m^−1^. This indicates that this increase is lower than the root-mean-square deviation of surface tension measurements.

### Surface Excess Concentration of Surfactants at the Water–Air Interface

Changes of water surface tension affected by the adsorption of the TX-100 and RL mixtures at the water–air interface depend on the density, composition and orientation of surfactant molecules in the surface layer. To show the correlation between those changes and the adsorption of surfactant mixtures, the Gibbs surface excess concentration of TX-100 (Fig. S7) and RL (Fig. S8) were calculated using the Gibbs isotherm adsorption equation [13, 22] (Eq. 7). For this purpose, the surface tension isotherms of TX-100 at constant RL concentration and vice versa were applied (Figs. 2, 3). At the concentrations of RL and TX-100 corresponding to their CMC or higher, it was difficult to determine the Gibbs surface excess concentration of these surfactants mathematically.

There are insignificant differences between the maximum TX-100 Gibbs surface excess concentrations at the lowest constant RL concentrations (Fig. S7) and the literature data determined for single TX-100 solutions under the same conditions [19]. Moreover, the maximum values of RL Gibbs surface excess concentration, at low constant TX-100 concentration (Fig. S8), are only marginally different from those determined by us for aqueous solutions of RL, which is equal to 2.01 × 10^−6^ mol·m^−2^ under the same conditions [18] but considerably lower than that obtained by Chen et al. [35] from the surface tension data at 30 °C in the UHQ-water system which is equal to 3.1 × 10^−6^ mol·m^−2^. The question arises why there are such discrepancies in the values of RL Gibbs surface excess concentration at the water–air interface obtained by us and Chen et al. [35]. This problem can be explained on the basis of Eq. 7. In the case of RL there are three possibilities (see the Supplementary Material). The first, is the case when RL is not dissociated in the bulk phase or in the surface region, then *n* in Eq. 7 is equal to 1. The second case refers to the state when RL is completely dissociated in the bulk phase and surface region, then in Eq. 7 *n* = 2. The most complicated is the third case, when RL can be present in the bulk phase and surface region in both dissociated and non-dissociated forms, because it is difficult to establish the accurate *n* values. The *n* value depends on the degree of dissociation of RL and can be in the range from 1 to 2 [35]. In our earlier studies [18] the RL Gibbs surface excess concentration was calculated using *n* = 2 in Eq. 7. That follows from our assumption that RL was completely dissociated in the bulk phase and surface region. In such a case, the surface area occupied by one surface active ion cannot be lower than the cross section of the RL molecule’s head. The area calculated by us is equal to 69.08 Å^2^ and corresponds to a value of the Gibbs surface excess concentration equal to 2.403 × 10^−6^ mol·m^−2^ [18]. If the RL molecules are dissociated in the surface region, there are strong repulsive interactions between the heads of the surface active ions. Therefore it is impossible to achieve the value of Gibbs surface excess concentration equal to 2.403 × 10^−6^ mol·m^−2^. On the other hand, the cross section of the RL tail, to a first approximation, is equal to 42 Å^2^. If the RL molecules are not dissociated in the surface region, attractive hydrophobic interactions between the head of one molecule and the tail of the other occur. In such a case, the minimal surface area occupied by the adsorbed RL molecules could be approximately equal to the average of 69.08 and 42 Å^2^ which is equal to 55.54 Å^2^. This area corresponds to the Gibbs surface excess concentration equal to 3 × 10^−6^ mol·m^−2^. This value is nearly the same as that obtained by Chen et al. [35]. As follows from our considerations, the RL Gibbs surface excess concentration is different, probably due to different conditions of surface tension measurements.

Knowing the Gibbs surface excess concentration of TX-100 at constant RL concentrations and vice versa (Figs. S7 and S8), it was possible to establish the total Gibbs surface excess concentration of the surfactant mixtures (Γ_12_) (Fig. S9 as an example). It appears that the shape of the isotherms of the Gibbs surface excess concentration of TX-100 and RL from their mixtures are similar to those of the individual surfactants [18, 19]. Indeed, the presence of RL in solution decreases the maximum adsorption of TX-100 and vice versa. It is difficult to describe the mutual effect of TX-100 and RL on their adsorption at the water–air interface based on the isotherms of the Gibbs surface excess concentrations of either surfactant at different constant concentrations of the other (Figs. S7 and S8). To obtain more information about the mutual influence of the surfactants on their adsorption, the total Gibbs surface excess concentration of TX-100 and RL was taken into account (Γ_12_ = Γ_1_ + Γ_2_) (Table 1). If *C*_1_ is equal to $C_{1}^{0}$(unsat.) and *C*_2_ to $C_{2}^{0}$(unsat.) respectively, [18, 19], then Γ_12_ = $\Gamma_{1}^{0}$ + $\Gamma_{2}^{0}$ (Table 1) (where $\Gamma_{1}^{0}$ is the Gibbs surface excess concentration of an individual TX-100 solution in the absence of RL and $\Gamma_{2}^{0}$ is the Gibbs surface excess concentration of an individual RL solution in the absence of TX-100). In the case when *C*_1_ is equal to $C_{1}^{0}$ and *C*_2_ to $C_{2}^{0}$ (both $C_{1}^{0}$ and $C_{2}^{0}$ are close to the concentration at which the saturated monolayer at the water–air interface starts to form), then Γ_12_ achieves its maximal value. This value is higher than the maximum Gibbs surface excess concentration of the single surfactants. On the other hand, when *C*_1_ is equal to $C_{1}^{0}$(sat.) and *C*_2_ to $C_{2}^{0}$(sat.), Γ_12_ < $\Gamma_{1}^{0}$ + $\Gamma_{2}^{0}$. It follows from the literature data that the maximal Gibbs surface excess concentration of an individual TX-100 solution ($\Gamma_{1}^{0(max)}$) is significantly higher than that of RL ($\Gamma_{2}^{0(max)}$) [18, 19]. The replacement of TX-100 molecules by the RL ions in the surface layer should decrease the maximal Γ_12_. On the other hand, hydrogen ions can be joined with the oxyethylene groups in the hydrophilic part of TX-100 and, in such a case, the density of the surface layer can increase. The possibility of joining the oxyethylene group is confirmed by the increase of pH of TX-100 and RL mixtures compared to the individual RL at the same concentration as in the mixture [18]. This effect causes the increase of the surface layer density; thus the increase of Γ_12_ is observed. These two phenomena are decisive for the value of Γ_12_. To explain more exactly the mutual effect of TX-100 and RL, the changes of Γ_12_ were considered as a function of the logarithm of the constant values of RL concentration (Fig. S10). It follows from Fig. S10 that Γ_12_ increases from the value close to $\Gamma_{1}^{0(max)}$ [19] to a maximum equal to 3.95 × 10^−6^ mol·m^−2^ (which corresponds to the first value of RL concentration at which it forms the saturated monolayer at the water–air interface in the absence of TX-100 [18]). Then it decreases to the value close to $\Gamma_{2}^{0(max)}$ [18]. Thus it can be stated that as the saturated TX-100 monolayer is formed and *C*_2_ is in the range from 0 to the first value corresponding to $C_{2}^{0}$(sat.), probably the ions of RL do not remove TX-100 molecules. These molecules are adsorbed only for the reason suggested above. The density of mixed monolayer increases to the maximal value. If *C*_2_ increases above the first point corresponding to $C_{2}^{0}$(sat.) [18], probably the replacement of TX-100 molecules by RL takes place and then Γ_12_ decreases because of the greater cross-sectional area of the RL molecule compared to that of TX-100. It should be also mentioned that there can be formed some aggregates of surfactants in the monolayer, which also affect the total Gibbs surface excess concentration of surfactant mixtures. The presented results indicate that RL plays a more important role than TX-100 in the mixed surface layer formation at the water–air interface.

### Composition of the Mixed Surface Layer

Changes of Γ_12_ should be reflected in the mixed layer composition. Therefore, taking into account both Γ_1_ and Γ_2_ (Figs. S7 and S8) and the limiting area of water, TX-100 and RL at the water–air interface [18, 19], the fractional area occupied by the molecules of TX-100 ($X_{1}^{0}$) and RL ($X_{2}^{0}$) at this interface as well as the mole fraction of individual surfactants in the mixed monolayer ($X_{1}^{S}$ and $X_{2}^{S}$) were determined (Eqs. 9, 10). Considering the relationship between the log_10_ $X_{1}^{0}$ and the log_10_ *C*_1_ or $X_{2}^{0}$ and *C*_2_ (Figs. S11 and S12) and that between the log_10_($X_{1}^{0}$ + $X_{2}^{0}$) and the log_10_ *C*_12_ (Figs. S13 and S14), it can be stated that these are of the Langmuir type [13, 23] only when the concentration of one surfactant in the mixture corresponds to its individual unsaturated monolayer (Fig. S11–S14) [18, 19].

Comparing the mole fractions of TX-100 ($X_{1}^{S}$) and RL ($X_{2}^{S}$) in the mixed monolayer to those in the bulk phase (1 − *α* and *α*, respectively), it can be stated that in the case when *C*_1_ and *C*_2_ are equal to $C_{1}^{0}$(unsat.) and $C_{2}^{0}$(unsat.), respectively, the RL mole fraction in the mixed surface layer is close to that resulting from independent adsorption of surfactants at the water–air interface. On the other hand, if the values of $C_{1}^{0}$ or $C_{2}^{0}$ correspond to $C_{1}^{0}$(sat.) or $C_{2}^{0}$(sat.), respectively, then the mole fraction of RL is higher than in the bulk phase and that of TX-100 lower (Figs. S15–S17). The difference between $X_{2}^{\text{S}}$ and *α* increases with increasing *C*_1_. This confirms our suggestion that the RL ion can adsorb at the interface along with TX-100 because of attractive interactions between the hydrophilic parts of these surfactants, due to binding the hydrogen ion of RL with the oxyethylene group in TX-100 [13]. The increase of the natural pH of the aqueous solution of TX-100 and RL mixtures compared to the pH of RL solutions in the absence of TX-100, at the same concentration as in this mixture, supports this suggestion. It was difficult to determine the composition of the surface layer in the case where the concentrations of both surfactants corresponded to their individual saturated monolayers. Therefore changes of the surface tension of the aqueous solution of surfactant mixtures were presented as a function of their concentration in the bulk phase, for example, for the solutions in which the mole fractions of surfactants in the bulk phase are equal to 0.5 (Fig. S18). Applying the Hua, Rosen and Rubingh method [13, 27, 28], mole fractions of both RL and TX-100 were determined using Eq. 13 and next the parameter of the intermolecular interactions in the monolayer (*β* ^*δ*^) from Eq. 14. The determined values of $X_{1}^{\text{S}}$ and $X_{2}^{\text{S}}$ indicate that RL’s tendency to adsorb at the water–air interface is greater than that of TX-100 because the values of $X_{2}^{\text{S}}$ (0.66–0.76) are higher than those of *α*. This explains why the maximal total Gibbs surface excess concentration decreases in the range of the concentration of both surfactants corresponding to their individual maximal surface excess concentration.

### Standard Gibbs Free Energy of Adsorption

Gibbs surface excess concentration should be reflected by the standard Gibbs free energy of adsorption ($\Delta G_{{_{\text{ads}} }}^{0}$) [22]. As mentioned earlier, the changes of the surface tension of the aqueous solutions of RL and TX-100, as a function of concentration of one component at constant concentration of the other, can be described by the Szyszkowski equation (Eq. 5) [13, 22], particularly if the concentration of one surfactant is lower than that corresponding to its saturated monolayer. In such a case, the constant *b* in this equation is equal to the constant in the Langmuir isotherm equation associated with $\Delta G_{{_{\text{ads}} }}^{0}$ (Eq. 16). $\Delta G_{{_{\text{ads}} }}^{0}$ was also determined using the Langmuir equation [13] modified by de Boer (Eq. 15) [29]. However, calculations of $\Delta G_{{_{\text{ads}} }}^{0}$ for the ionic surfactant from this equation are not unambiguous, because, for a 1:1 type ionic surfactant, some researchers applied 2*RT* but others *RT*. It should be taken into account that the Gibbs surface excess concentration of the ionic surface active agent is calculated from the Gibbs isotherm equation in which 2*RT* is used on the assumption that the ionic surfactant is completely dissociated in the bulk phase and surface region (see Supplementary Material). The Gibbs surface excess concentration of a surface active ion and the area occupied by it were determined using this assumption; therefore, in the Langmuir equation, *RT* was used.

As follows from the calculations, $\Delta G_{{_{\text{ads}} }}^{0}$ values are constant in the range of one surfactant concentration corresponding to its unsaturated monolayer in the absence of another (Figs. S19 and S20). This means that there are no interactions between the TX-100 and RL molecules or that these interactions only insignificantly influence on $\Delta G_{{_{\text{ads}} }}^{0}$. If these constant values of $\Delta G_{{_{\text{ads}} }}^{0}$ are taken into account at a given constant concentration of one surfactant, it can be stated that there is the greatest tendency to adsorb RL at *C*_1_ corresponding to $C_{1}^{0}$(sat.) (Fig. S21). This confirms our suggestion that H_3_O^+^ ions may be bonded with the oxyethylene groups of TX-100, causing that it behave as a cationic surfactant and allowing it to interact by attractive electrostatic forces with the RL ions. Consequently, the RL ions can be adsorbed both with TX-100 and individually. For this reason, Γ_12_ is higher than those for the single surfactants in the range of concentrations from 0 to *C* ^0^(unsat.) in the bulk phase. At *C*_1_ corresponding to $C_{1}^{0}$(unsat.) [19], the standard Gibbs free energy for RL adsorption is constant and close to its energy in the absence of TX-100 [18] (if it is calculated from the Langmuir equation using *RT*). On the other hand, the standard Gibbs free energy of TX-100 adsorption in the concentration range of *C*_2_ corresponding to $C_{2}^{0}$(unsat.) [18] changes insignificantly. This confirms that in the range of TX-100 and RL concentrations corresponding to their individual unsaturated monolayer, independent adsorption of these surfactants takes place. The values of $\Delta G_{{_{\text{ads}} }}^{0}$ calculated from the *b* constant in the Szyszkowski equation [13, 22] are different from those obtained from the Langmuir equation. This results from the fact that the constant *b* is obtained from best fits of the surface tension of the aqueous solutions of TX-100 and RL mixtures to those measured in the range of concentrations of one surfactant from 0 to the CMC of the mixture, at a given constant concentration of the other.

### Critical Micelle Concentration (CMC) and Standard Gibbs Free Energy of Micellization

**Fig. 4:**
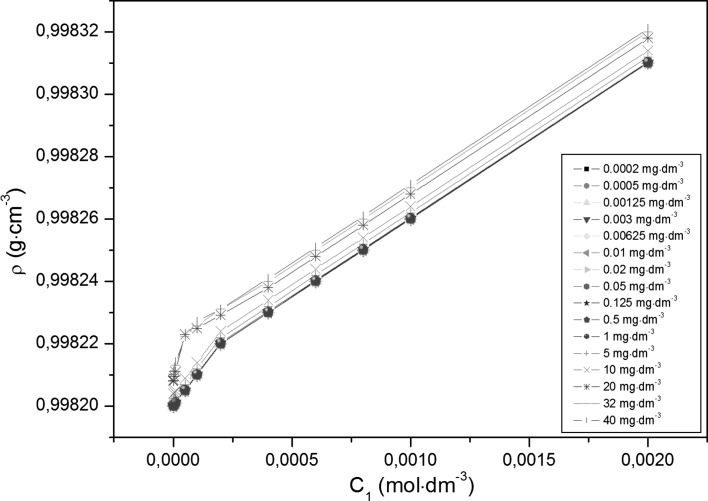
A plot of the density (*ρ*) of the aqueous solutions of TX-100 and RL mixtures at constant RL concentration versus the TX-100 concentration in the bulk phase (*C*_1_). *Curves 1–16* as in Fig. 1

**Fig. 5:**
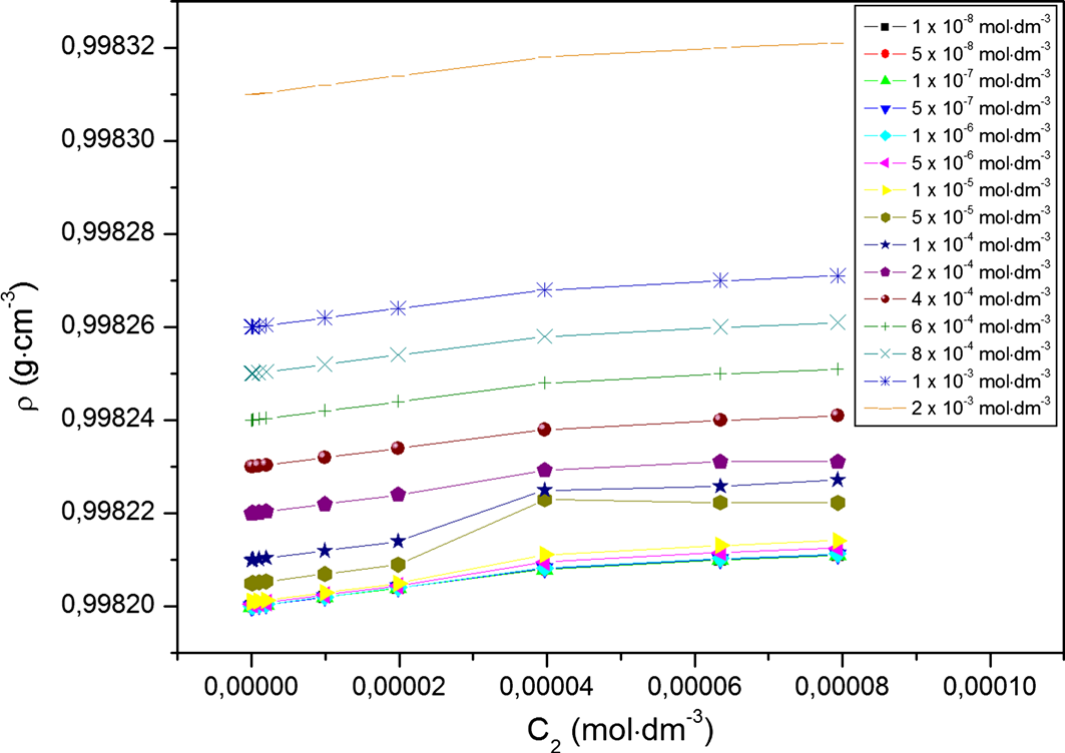
A plot of the density (*ρ*) of the aqueous solutions of TX-100 and RL mixtures at constant TX-100 concentration versus the RL concentration in the bulk phase (*C*_2_). *Curves 1–15* as in Fig. 2

**Fig. 6:**
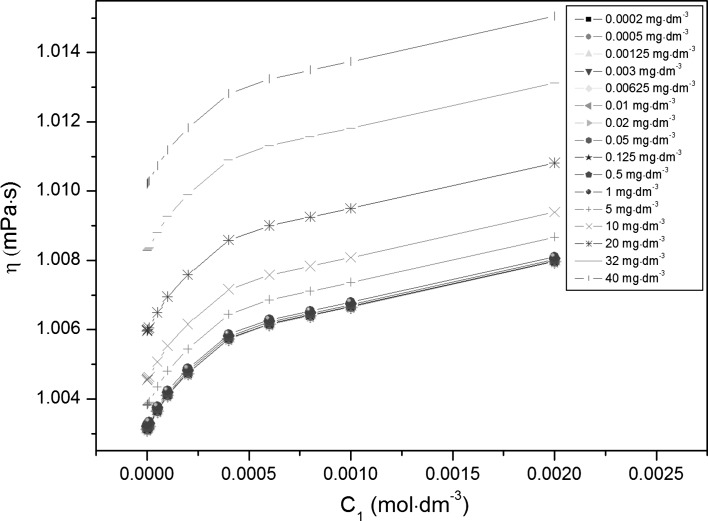
A plot of the viscosity (*η*) of the aqueous solutions of TX-100 and RL mixtures at constant RL concentration versus the TX-100 concentration in the bulk phase (*C*_1_). *Curves 1–16* as in Fig. 1

**Fig. 7:**
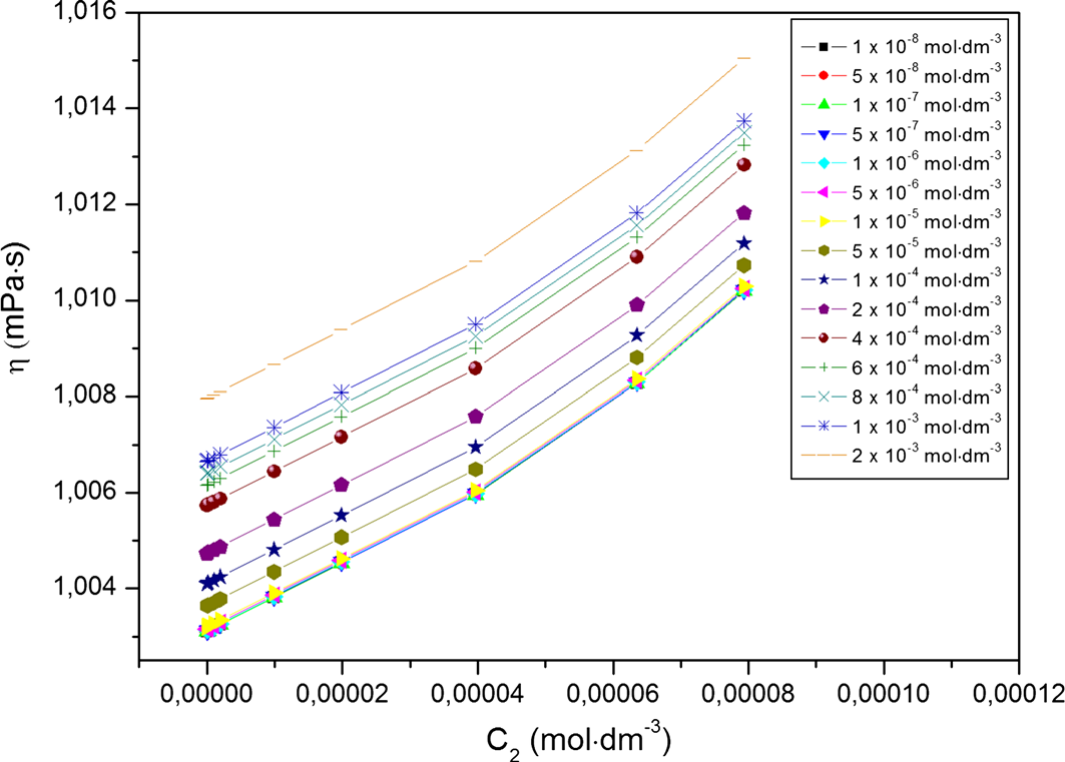
A plot of the viscosity (*η*) of the aqueous solutions of TX-100 and RL mixtures at constant TX-100 concentration versus the RL concentration in the bulk phase (*C*_2_). *Curves 1–15* as in Fig. 2

Adsorption of TX-100 and RL mixtures at the water–air interface is also connected with their aggregation in the bulk phase, which starts at the critical micelle concentration (CMC) and can be determined from the measurements of many physicochemical properties [13]. However, the experimental CMC values can depend on the method of their determination. The CMC values of TX-100 and RL mixtures (*CMC*_12_) determined from the dependence between the surface tension and the log_10_ of RL or TX-100 concentration (Figs. 2, 3) are slightly higher than those determined on the basis of the density (Figs. 4, 5) and dynamic viscosity data (Figs. 6, 7). Considering the changes of *CMC*_12_ as a function of RL mole fraction in the bulk phase (*α*), it can be stated that there is negative deviation of *CMC*_12_ from ideal behavior for the RL and TX-100 mixtures in the range of *α* from 0 to 0.5 (Fig. S22). However, the changes of *CMC*_12_ as a function of log_10_ *C*_1_ are different from those as a function of log_10_ *C*_2_ (Fig. S23). On the other hand, at constant *C*_1_ equal to $C_{1}^{0}$(unsat.), the values of *CMC*_12_ are practically constant and close to *CMC*_2_ [18]. At *C*_1_ higher than *CMC*_1_ [23], an increase of *CMC*_12_ is observed. However, it was difficult to determine values of *CMC*_12_ at high concentrations of the surfactants. The values obtained probably indicate that there is some transformation of the micelles. In the case of the constant RL concentration *CMC*_12_ decreases from *CMC*_1_ [22] to *CMC*_2_ [18].

The tendency to form the mixed micelle of RL and TX-100 depends on the standard Gibbs free energy of micellization ($\Delta G_{\text{mic}}^{0}$). In the literature it is possible to find different approaches to determine this energy [22, 32] but in the case of the mixtures of ionic and non-ionic surfactants, when the concentration of one surfactant changes from 0 to its CMC, it is very difficult to choose a proper method to determine $\Delta G_{\text{mic}}^{0}$. As mentioned above, it is probable that the H_3_O^+^ ions can bind with the oxyethylene groups in the TX-100 molecule. If such bonding takes place in the mixed micelles, the charge of RL may be neutralized and the mixed micelles can be treated as non-ionic. If so, for calculation of $\Delta G_{\text{mic}}^{0}$ the following equation can be applied [13, 36]:

26 $$ \Delta G_{\text{mic}}^{0} = RT\ln \frac{CMC}{\varpi } $$

However, it should be remembered that our assumption is more realistic in the case when RL is added to the solution of TX-100 than vice versa. Our calculations show that there is a negative deviation of $\Delta G_{\text{mic}}^{0}$ from that for ideal mixing over the whole range of *α* (Fig. S25). The standard Gibbs free energy of micellization for the surfactant mixtures should result from the standard Gibbs free energy of ideal mixture for micellization and the excess Gibbs free energy of non-ideal mixing of surfactants in the micelle (Eq. 23). In fact, the sum of the standard Gibbs free energy of micellization, calculated on the basis of *CMC*_1_ and *CMC*_2_ and the Gibbs free energy of non-ideal mixing of RL and TX-100 is close to the standard Gibbs free energy of micellization of RL and TX-100 mixtures calculated from Eq. 26.

### Synergetic Effect in the Reduction of Water Surface Tension and Micelle Formation

Calculations based on the Hua, Rosen and Rubingh methods [13, 27, 28] indicate that there is a synergetic effect in the reduction of water surface tension by the TX-100 + RL mixtures, because the parameter of the intermolecular interactions (*β* ^*δ*^) is negative and its absolute value is higher than that of the natural logarithm of the ratio of RL to TX-100 concentration at the same surface tension of their aqueous solutions (|*β* ^*δ*^| > |ln $C_{2}^{0}$/$C_{1}^{0}$|) [13] (Table 2).

**Table 2:** The synergetic effect of the TX-100 and RL mixtures in the reduction of the water surface tension and mixed micelle formation

*γ*_*LV*_[mN·m^−1^]	*β* ^*δ*^	ln (*C*_2_^o^/*C*_1_^o^)	Synergetic effect
Synergetic effect in the reduction of the water surface tension			
55	−2.3538	−1.4696	+
50	−2.2470	−1.6765	+
45	−2.2616	−1.9261	+
40	−2.2804	−2.1050	+
35	−2.2943	−2.2814	+

*α*	*β* ^M^	ln (*CMC*_2_/*CMC*_1_)	Synergetic effect
Synergetic effect in the mixed micelle formation			
1.50 × 10^−6^	−10.0077	−1.7113	+
3.74 × 10^−6^	−9.0387	−1.7113	+
9.39 × 10^−6^	−8.1272	−1.7113	+
2.34 × 10^−5^	−7.7355	−1.7113	+
5.30 × 10^−5^	−7.7834	−1.7113	+
8.98 × 10^−5^	−7.7216	−1.7113	+
1.90 × 10^−4^	−7.3135	−1.7113	+
4.99 × 10^−4^	−6.5303	−1.7113	+
0.00104	−5.9174	−1.7113	+
0.00567	−4.3157	−1.7113	+
0.012	−4.2500	−1.7113	+
0.0927	−3.6475	−1.7113	+
0.207	−2.4896	−1.7113	+
0.256	−2.0780	−1.7113	+
0.337	−1.3760	−1.7113	−
0.447	−0.4402	−1.7113	−
0.517	−0.0980	−1.7113	−
0.533	−0.0770	−1.7113	−
0.548	−0.0560	−1.7113	−
0.864	−1.4453	−1.7113	−
0.909	−2.7170	−1.7113	+
0.981	−4.4100	−1.7113	+
0.991	−4.6310	−1.7113	+
0.998	−4.7610	−1.7113	+
0.999	−4.8379	−1.7113	+

+ |*β* ^*δ*^| > | ln ($$ C_{2}^{0} $$/$$ C_{1}^{0} $$)|, − |*β* ^*δ*^| < | ln ($$ C_{2}^{0} $$/$$ C_{1}^{0} $$)|

+ |*β* ^M^| > | ln (*CMC*_2_/*CMC*_1_)|, − |*β* ^M^| < | ln (*CMC*_2_/*CMC*_1_)|

To establish whether the synergetic effect in the mixed micelle formation takes place, the Hua, Rosen and Rubingh method [13, 27, 28] was used. By means of this method, the compositions of mixed micelles, parameter of intermolecular interactions in the mixed micelles (*β* ^M^) and activity coefficients of the components were determined (Eqs. 21, 22, 23, 25) as well as the conditions that should be satisfied for the synergetic effect were evaluated. It proved that in all cases $X_{2}^{\text{M}}$ is higher than *α* and *β* ^M^ assumes negative values (Fig. S24).

This means that the first condition for the synergetic effect in the mixed micelle formation is satisfied, however, the second one (|*β* ^*M*^| > |ln *CMC*_2_/*CMC*_1_|) [13] is not fulfilled in the range of *α* from 0.337 to 0.864 (Table 2).

Apart from the regular solution theory proposed by Hua, Rosen and Rubingh used to establish a synergetic effect in the mixed micelle formation, it is also possible to find in the literature the theory proposed by Bergström and Eriksson [37] which is based on the Poisson–Boltzmann distribution. For the systems of non-ionic and ionic surfactant mixtures they derived an equation to predict the CMC which has the form:

27 $$ CMC_{12} \left( {X_{2} } \right) = \left( {X_{2} } \right)^{2} \exp \left( {1 - X_{2} } \right)CMC_{2} + \left( {1 - X_{2}^{{}} } \right)\exp (1 - X_{2} )CMC_{1}. $$

**Fig. 8:**
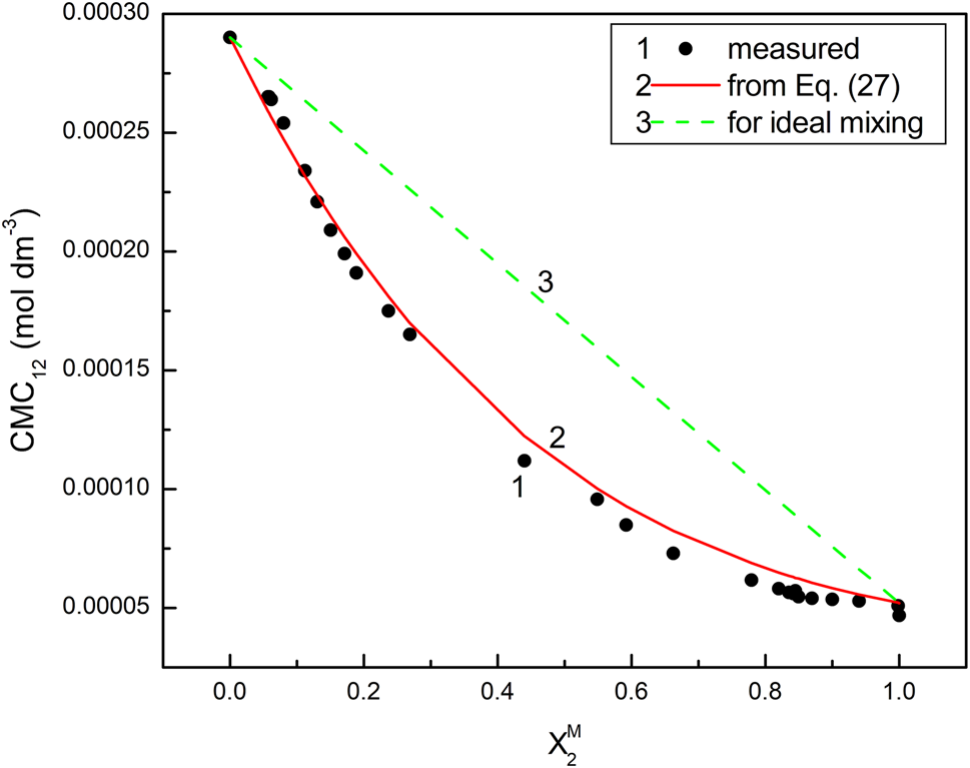
A plot of the critical micelle concentration of TX-100 and RL mixture (CMC12) versus RL mole fraction in the mixed micelle (X2M). *Points 1* correspond to the values determined experimentally, *curve 2* to the value calculated from Eq. 27 and *curve 3* to the value for ideal mixture

Using the mole fraction of RL in the mixtures determined on the basis of the Hua, Rosen and Rubingh theory in Eq. 27, the calculated values of *CMC*_1_ and *CMC*_2_ are very close to those obtained from the surface tension isotherm (Fig. 8). This confirms the existence of the synergetic effect in the mixed micelle formation by TX-100 and RL; however, contrary to the Hua, Rosen and Rubingh method this effect is evident in the whole range of TX-100 and RL mixture composition.

### Apparent and Partial Molar Volumes of RL and TX-100 in the Aqueous Solutions of their Mixtures

It should be expected that the mixed micelle formation can be reflected by the changes of the apparent (*φ*_*V*_) and partial molar volumes ($\overline{V}_{\text{m}}$) [30, 31] (Eqs. 18, 19) (Figs. S26–S29). The changes of *φ*_*V*_ and $\overline{V}_{\text{m}}$ values of RL at a given constant TX-100 concentration (Figs. S27 and S29) only slightly depend on the value of this concentration. In the range of RL concentration in which it is present in the monomeric form in solution, the apparent molar volume is constant and insignificantly decreases when the micelles are formed. Contrary to the apparent molar volume, the partial one increases linearly as a function of RL concentration in the bulk phase but practically does not depend on the TX-100 concentration. The changes of RL partial molar volume occur in the range from 406 to 472 mL· mol^−1^. The partial molar volume of TX-100 (Fig. S28) also increases linearly as a function of its concentration in the solution; however, in this case the effect of RL concentration is evident. This value changes from 579 to 609 mL·mol^−1^. The apparent molar volume of TX-100 (Fig. S26) increases in the range of its concentration in which it is present in the solution in the monomeric form [23]. It is constant when micelles are present in equilibrium with the monomeric form of TX-100.

Changes of apparent and partial molar volumes can be explained from the size of surfactant molecules and the average distances between the surfactant molecules and water in solution as well as surfactant molecules in the micelles. It is reasonable to assume that the average distance between the hydrophilic part of surfactants and water in the monomeric and aggregated forms is nearly the same but the average distance between the hydrophobic part and water or between the hydrophobic parts of surfactants in micelles can be different. Taking into account the length of the bonds between the atoms in the RL and TX-100 molecules and the angle between their bonds, the volumes of particular groups in the RL and TX-100 molecules were assumed to be equal to the cubes at proper sizes. Assuming that the minimal average distance between the moieties cannot be shorter than 1.56 Å and the maximal distance is the same as in the hydrocarbon media (2 Å), the molar volumes of TX-100 and RL were calculated. For RL the molar volumes at the average distance 1.56 and 2 Å are equal to 407.15 and 469.5 mL·mol^−1^ and for TX-100 557.4 and 605 mL·mol^−1^, respectively. This proved that the changes of partial and apparent molar volumes of TX-100 and RL are in the range of volumes determined for the minimal and maximal average distances between the hydrocarbon part and water as well as between the hydrocarbon parts of surfactants.

## Conclusions

The analysis of the results obtained from the surface tension, density and viscosity measurements indicates that:

If the concentrations of TX-100 and RL in the mixture are lower than those corresponding to their individual saturated monolayers at the water–air interface, then there is independent adsorption of surfactants and the changes of the aqueous solution surface tension as a function of their concentration can be predicted by the Joos or Fainerman and Miller equations and described by that of Szyszkowski.

The maximal Gibbs surface excess concentrations of TX-100 and RL mixtures at the water–air interface are found when their concentrations in the bulk phase correspond to those at which the saturated monolayer of the individual surfactants at this interface starts to form.

The synergic effect occurs in the process of mixed micelle formation by TX-100 and RL and in the reduction of the water surface tension.

The mole fraction of RL in the mixed monolayer at the water–air interface and in the mixed micelles is higher than in the bulk phase, which is probably due to electrostatic interactions between the hydrophilic part of TX-100 with the bound H_3_O^+^ group and COO^−^ ion in the hydrophilic part of RL.

The critical micelle concentrations of TX-100 and RL mixtures can be predicted, to a first approximation, from the Bergström and Eriksson equation.

Changes of apparent and partial molar volumes of RL and TX-100 were explained based on theoretical calculations of RL and TX-100 molecular sizes considering different average distances between their hydrophobic part and water molecules or between them.

## Electronic supplementary material

Below is the link to the electronic supplementary material.

Supplementary material 1 (DOC 1185 kb)
