# Supplementary material for: Composition of Surface Layer at the Water–Air Interface and Micelles of Triton X-100 + Rhamnolipid Mixtures
Source: J Solution Chem. 2017 Jun 15;46(6):1251–71. doi: 10.1007/s10953-017-0642-7 (PMC5488207; doi:10.1007/s10953-017-0642-7)
Supplement: Supplementary file 1 — Supplementary material 1 (DOC 1185 kb) [file 10953_2017_642_MOESM1_ESM.doc]

**COMPOSITION OF SURFACE LAYER AT WATER-AIR INTERFACE AND MICELLE OF TRITON X-100 AND RHAMNOLIPID MIXTURE**

DIANA MAŃKO, ANNA ZDZIENNICKA*, AND BRONISŁAW JAŃCZUK

*Department of Interfacial Phenomena, Faculty of Chemistry, Maria Curie-Skłodowska University, Maria Curie-Skłodowska Sq. 3, 20-031 Lublin, Poland*

Running title: Composition of surface layer

*To whom correspondence should be addressed

phone (48-81) 537-56-70

fax (48-81) 533-3348

e-mail aniaz@hektor.umcs.lublin.pl

**Gibbs isotherm equation for multicomponents system**

For the multicomponents system the Gibbs isotherm equation can be written in the form [1, 2]:

$\Gamma_{i}=\frac{-a_{i}}{nRT}\left(\frac{d\gamma}{da_{i}}\right)_{T,p,n_{j\neq i}}$ (1)

where $\Gamma_{i}$ is the Gibbs surface excess concentration of the *i* component and $a_{i}$ is its activity in the bulk phase, $\gamma$ is the surface tension of solution, $T$ is the absolute temperature, $R$ is the gas constant, $p$ is the pressure.

This equation can be derived from the Gibbs-Duhem one [3]. At *T* and *p =* const. for surface region this equation assumes the following form:

$Ad\gamma=-\sum_{i=1}^{\alpha} n_{i}^{s}d\mu_{i}^{s}$ (2)

where $\alpha$ is the number of components in the system, $A$ is the Gibbs interface area, $n_{i}^{s}$ is the excess of the moles of *i* component in the surface region and $\mu_{i}^{s}$ is the chemical potential of *i* component in the surface region.

As it is commonly known in the equilibrium state $\mu_{i}^{s}=\mu_{i}$($\mu_{i}$ is the chemical potential of *i* component in the bulk phase). Taking into account that:

$\mu_{i}=\mu_{i}^{o}+RT\ln a_{i}$ (3) at *T* = const. and *p* = const. from Eq. (2) we obtain:

$Ad\gamma=-RT\sum_{i=1}^{\alpha} n_{i}^{s}d\ln a_{i}$ (4) Of course, each component of the system must have the same form in both phases. It means that in the equilibrium state if one molecule or one ion of a given substance transfers from the bulk phase to the surface region, then the other one should transfer from the surface region to the bulk phase and during this transfer none of them changes its form.

In the aqueous solution of rhamnolipid (RL) and Triton X-100 (TX-100) mixture there are the following components: H_2_O, TX-100, RL, RL^-^, H_3_O^+^ and OH^-^. Taking into account that [H_3_O^+^][ OH^-^] is constant and choosing the Gibbs surface area in such a way that the excess of H_2_O molecules is equal to zero from Eq. (4) we obtain:

$Ad\gamma=-RT\left[n_{1}^{s}d\ln a_{1}+n_{2}^{s}d\ln a_{2}+n_{3}^{s}d\ln a_{3}+n_{4}^{s}d\ln a_{4}\right]$ (5)

where subscripts 1, 2, 3 and 4 correspond to TX-100, RL, RL^-^ and H_3_O^+^, respectively.

For the diluted solution the activity of its components gets closer to the mole fraction which can be expressed as equal to $C_{i}/\omega$ ($\omega$ - the number of water moles in 1 dm^3^, $C_{i}$ - the concentration of *i* component in moledm^-3^). In such a case Eq. (5) assumes the form:

$Ad\gamma=-RT\left[n_{1}^{s}d\ln C_{1}+n_{2}^{s}d\ln C_{2}+n_{3}^{s}d\ln C_{3}+n_{4}^{s}d\ln C_{4}\right]$ (6)

If the concentration of one component changes and the other is constant, then Eq. (6) assumes the form of Eq. (1).

In the case when the RL concentration is constant and TX-100 does not influence on the degree of RL dissociation, then in Eq. (1) $n=1$. When the TX-100 concentration is constant there are three possibilities of Eq. (6) solution. The first one is dealing with the case when RL is not dissociated in the bulk phase and surface region. Then$n$ in Eq. (1) is also equal to 1. The second case refers to state when RL is completely dissociated in the bulk phase and surface region, then in Eq. (1) $n=2$. The most complicated is the third case when RL can be presented in the bulk phase and in the surface region both in the dissociated and non-dissociated forms. Thus to solve Eq. (6) knowledge of dissociation degree of RL in the bulk phase and surface region is necessary. In this case $n$ in Eq. (1) can be changed from 1 to 2. In practice, on the basis of our measurements this equation can be solved only in two cases. The first case is when RL is not dissociated in the bulk phase and surface region and the second one when RL is completely dissociated in both these phases.

**References**

[1] J.M. Rosen, Surfactants and Interfacial Phenomena, third ed., Wiley Interscience, New York, 2004.

[2] A.W. Adamson, A.P. Gast, Physical Chemistry of Surfaces, sixth ed.,
Wiley-Interscience, New York, 1997.

[3] J. Ościk, Adsorption, 1982.

**
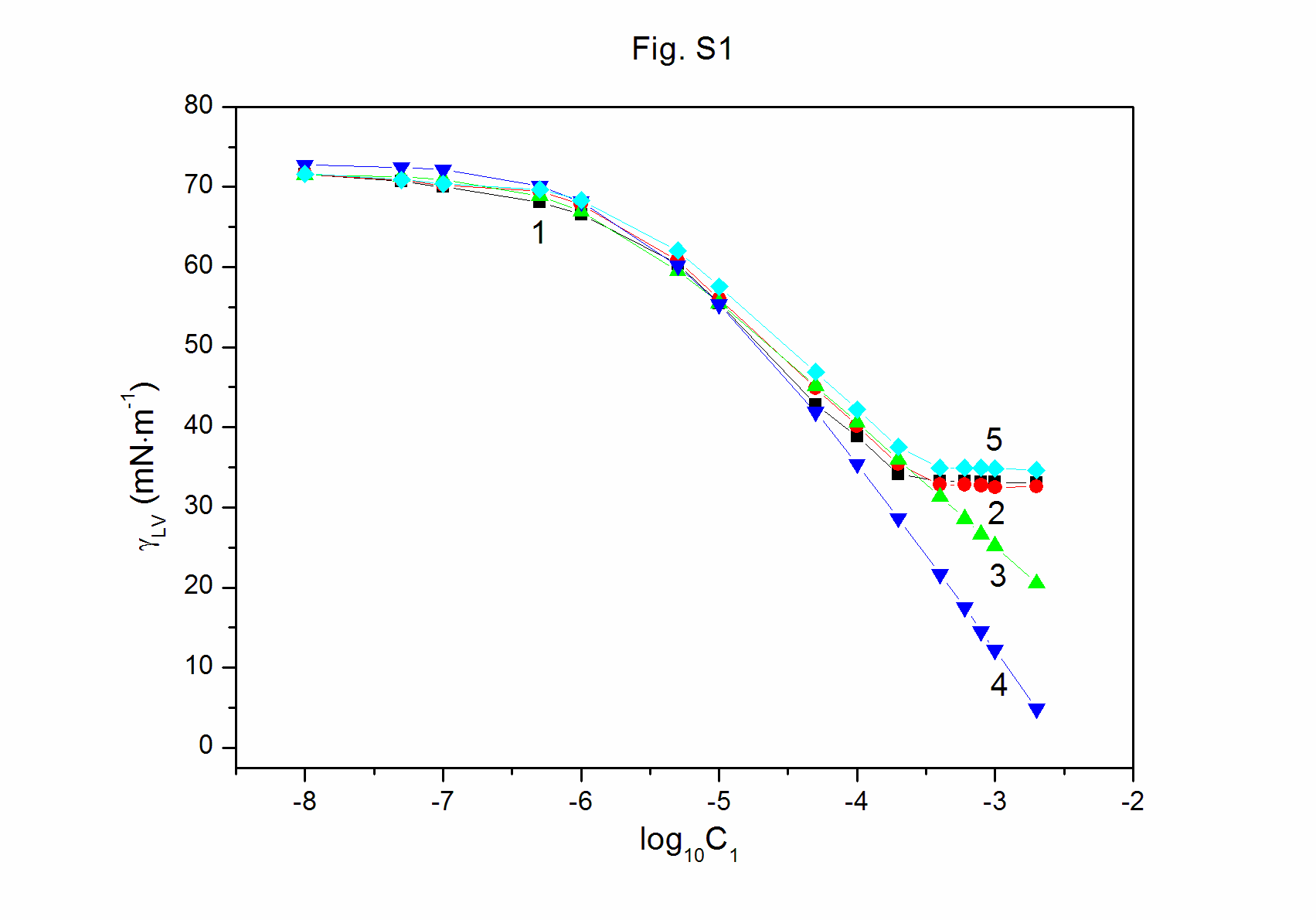
**

**Fig. S1.** A plot of the surface tension ($\gamma_{LV}$) of aqueous solution of TX-100 and RL mixture at the constant RL concentration (0.0002 mgdm^-3^; 3.97 x 10^-10^ moldm^-3^) vs. the logarithm_10_ of TX-100 concentration in the bulk phase ($C_{1}$). Curve 1 corresponds to the measured values of ($\gamma_{LV}$), curves 2 - 5 to those calculated from Eqs. (1), (5), (2) and (6), respectively.


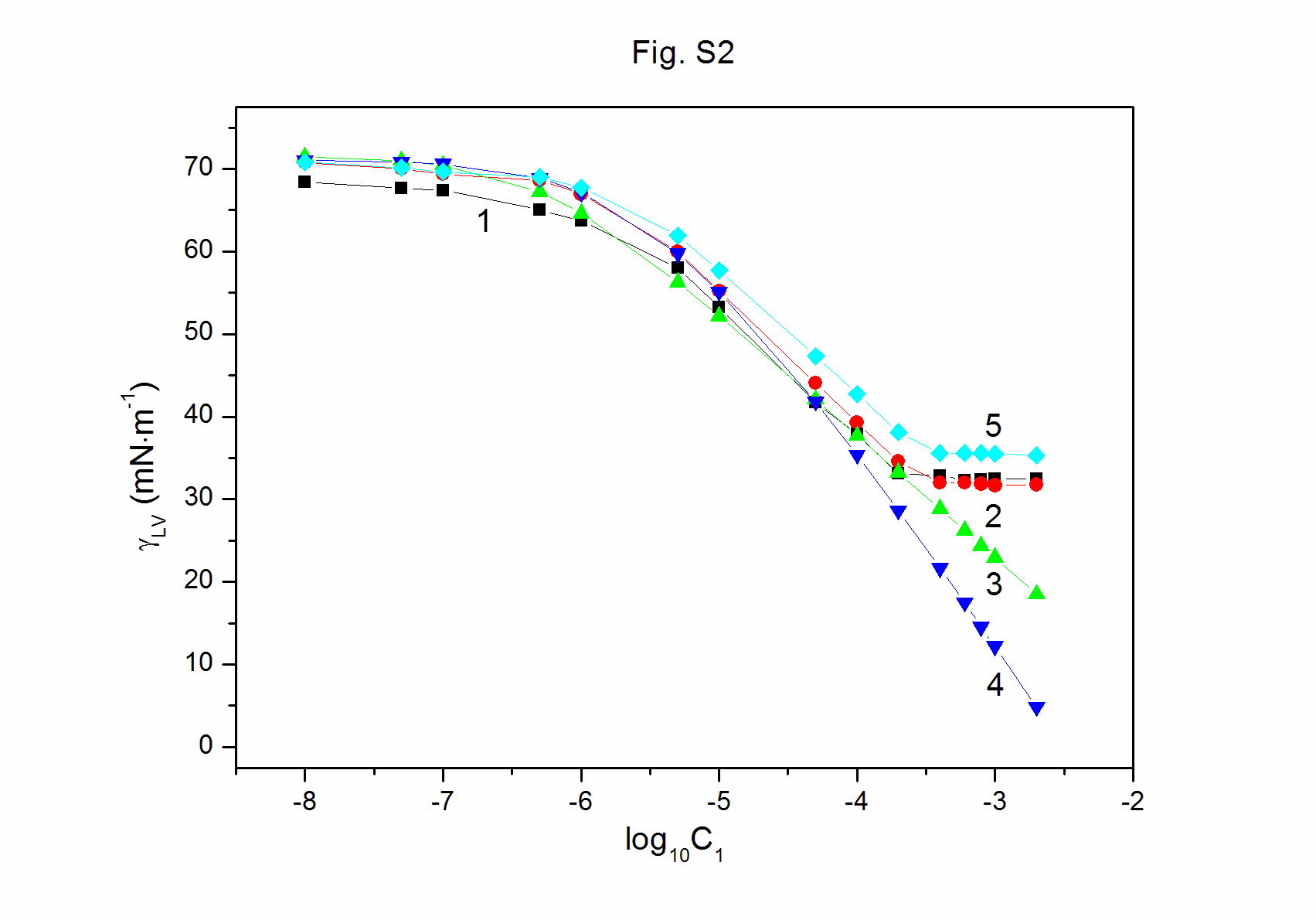


**Fig. S2.** A plot of the surface tension ($\gamma_{LV}$) of aqueous solution of TX-100 and RL mixture at the constant RL concentration (0.05 mgdm^-3^; 9.92 x 10^-8^ moldm^-3^) vs. the logarithm_10_ of TX-100 concentration in the bulk phase ($C_{1}$). Curve 1 corresponds to the measured values of ($\gamma_{LV}$), curves 2 - 5 to those calculated from Eqs. (1), (5), (2) and (6), respectively.

**
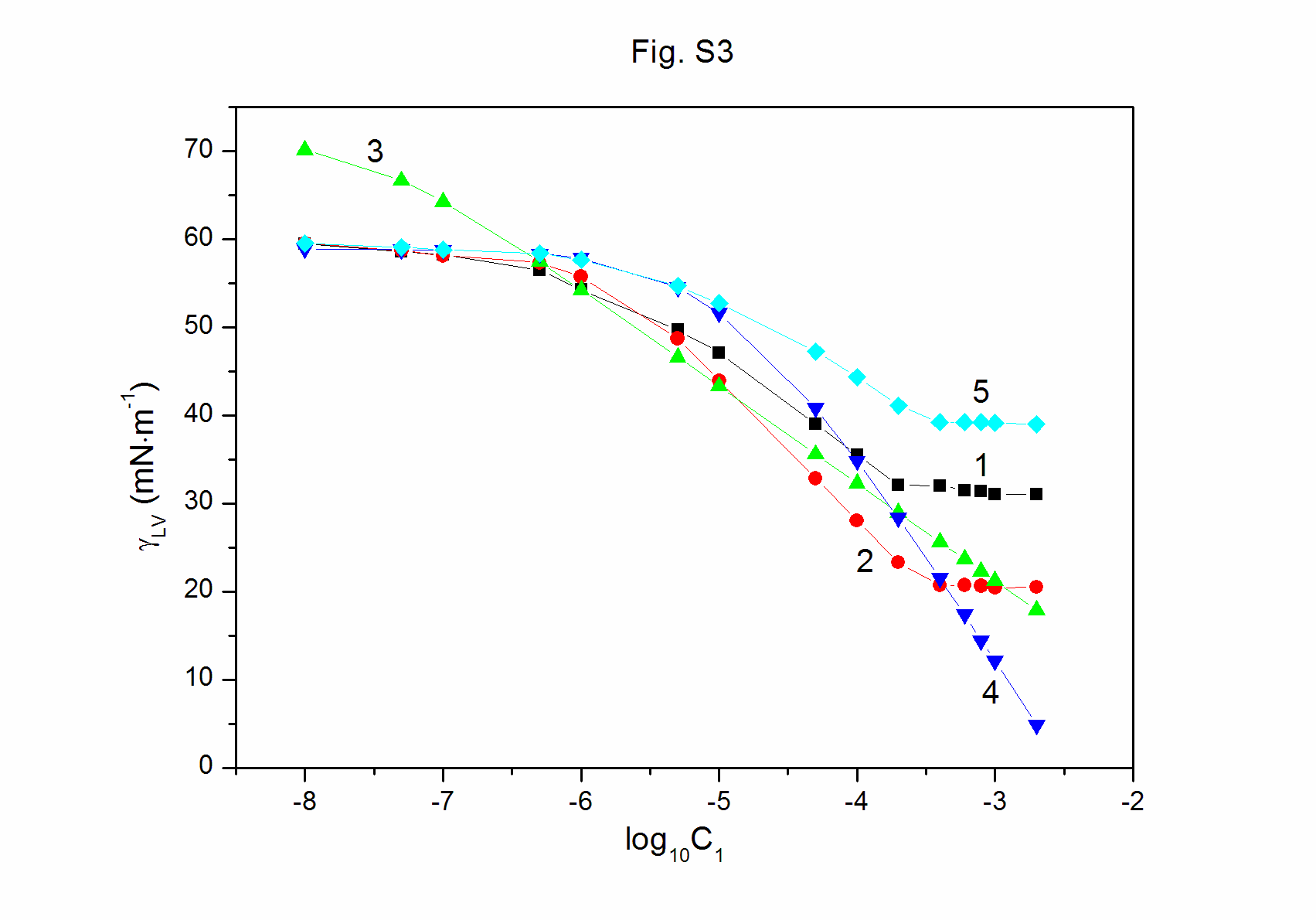
**

**Fig. S3.** A plot of the surface tension ($\gamma_{LV}$) of aqueous solution of TX-100 and RL mixture at the constant RL concentration (1 mgdm^-3^; 1.98 x 10^-6^ moldm^-3^) vs. the logarithm_10_ of TX-100 concentration in the bulk phase ($C_{1}$). Curve 1 corresponds to the measured values of ($\gamma_{LV}$), curves 2 - 5 to those calculated from Eqs. (1), (5), (2) and (6), respectively.


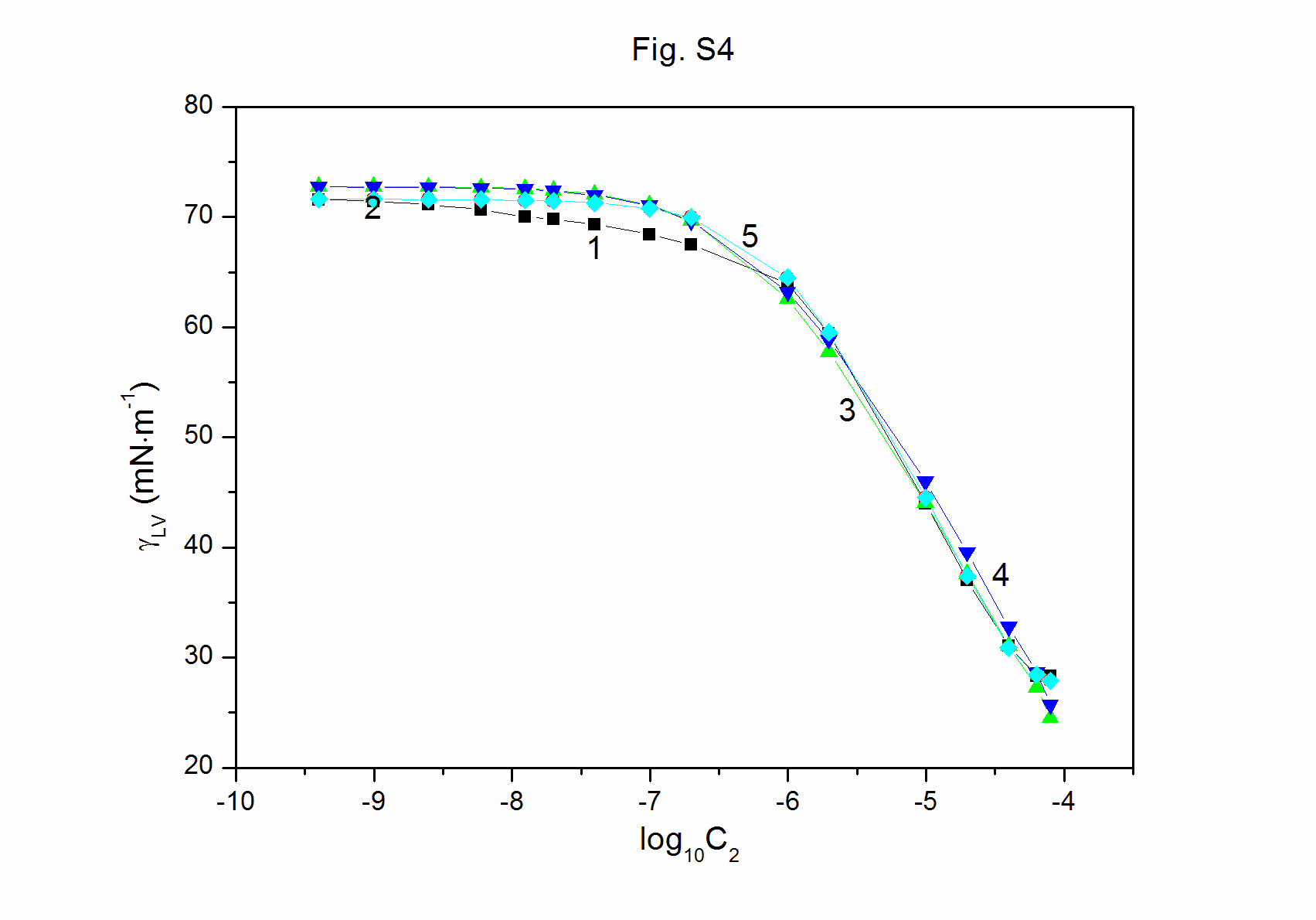


**Fig. S4.** A plot of the surface tension ($\gamma_{LV}$) of aqueous solution of TX-100 and RL mixture at the constant TX-100 concentration (1 x 10^-8^ moldm^-3^) vs. the logarithm_10_ of RL concentration in the bulk phase ($C_{2}$). Curve 1 corresponds to the measured values of ($\gamma_{LV}$), curves 2 - 5 to those calculated from Eqs. (1), (5), (2) and (6), respectively.

**
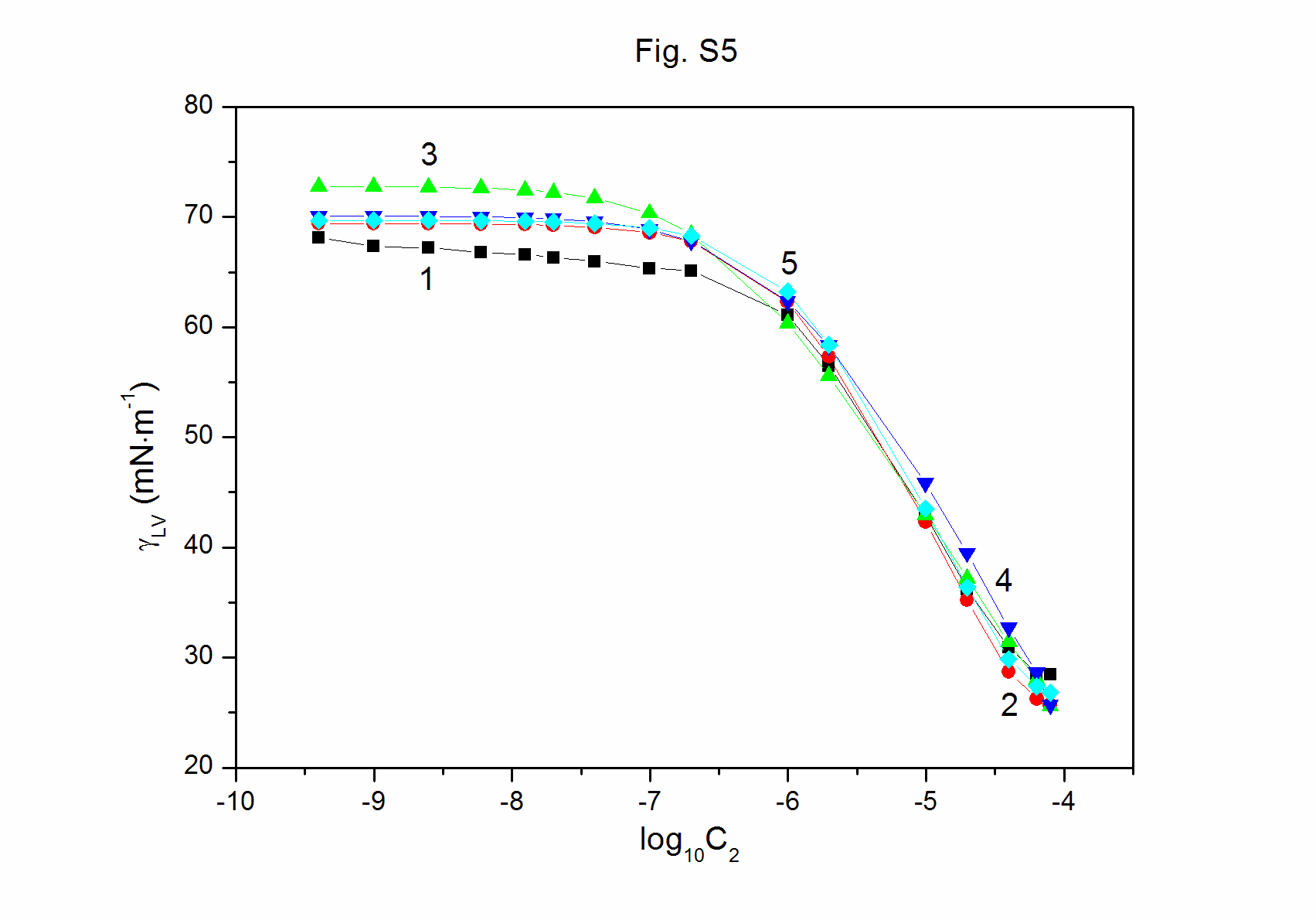
**

**Fig. S5.** A plot of the surface tension ($\gamma_{LV}$) of aqueous solution of TX-100 and RL mixture at the constant TX-100 concentration (5 x 10^-7^ moldm^-3^) vs. the logarithm_10_ of RL concentration in the bulk phase ($C_{2}$). Curve 1 corresponds to the measured values of ($\gamma_{LV}$), curves 2 - 5 to those calculated from Eqs. (1), (5), (2) and (6), respectively.

**
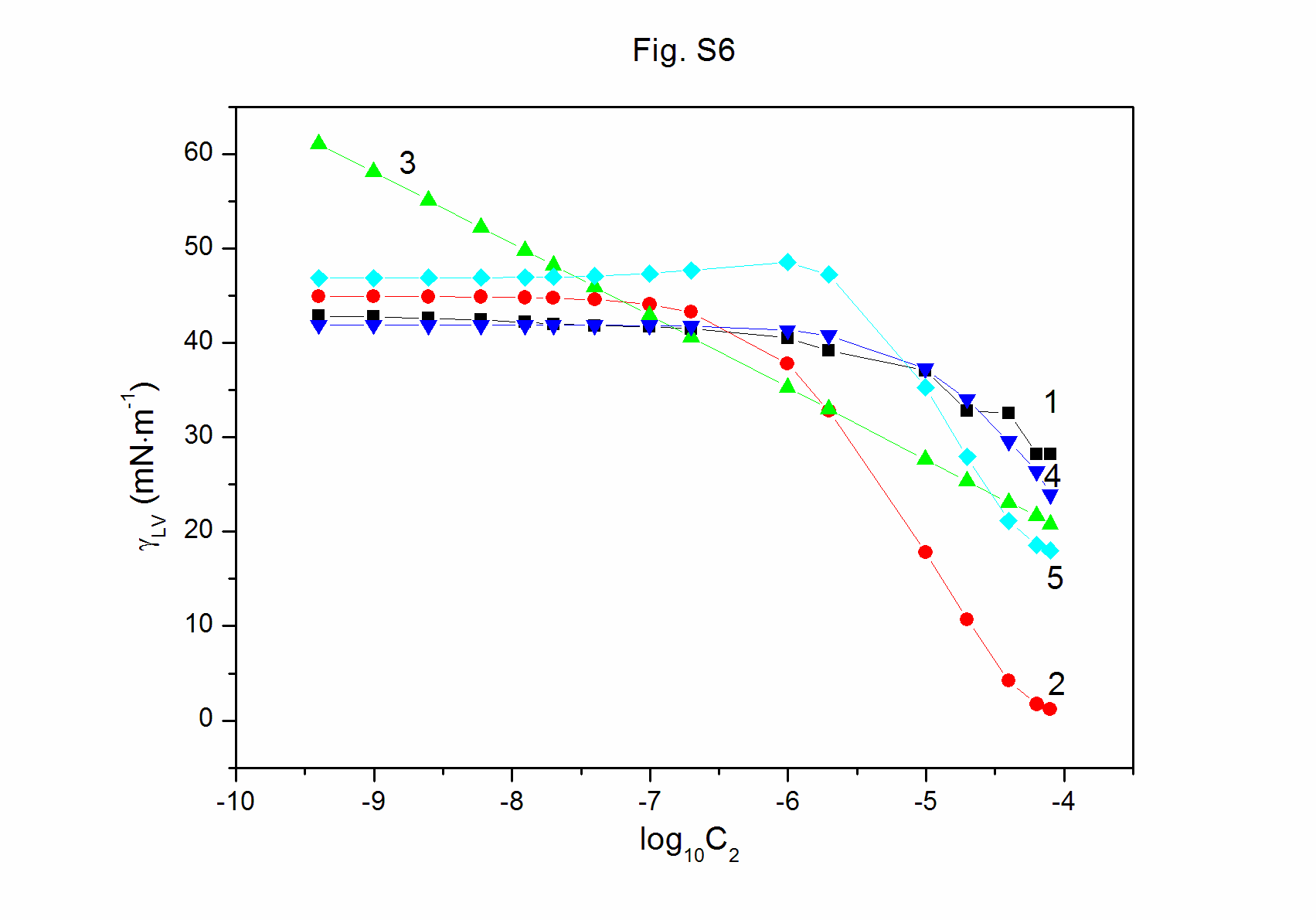
**

**Fig. S6.** A plot of the surface tension ($\gamma_{LV}$) of aqueous solution of TX-100 and RL mixture at the constant TX-100 concentration (5 x 10^-5^ moldm^-3^) vs. the logarithm_10_ of RL concentration in the bulk phase ($C_{2}$). Curve 1 corresponds to the measured values of ($\gamma_{LV}$), curves 2 - 5 to those calculated from Eqs. (1), (5), (2) and (6), respectively.


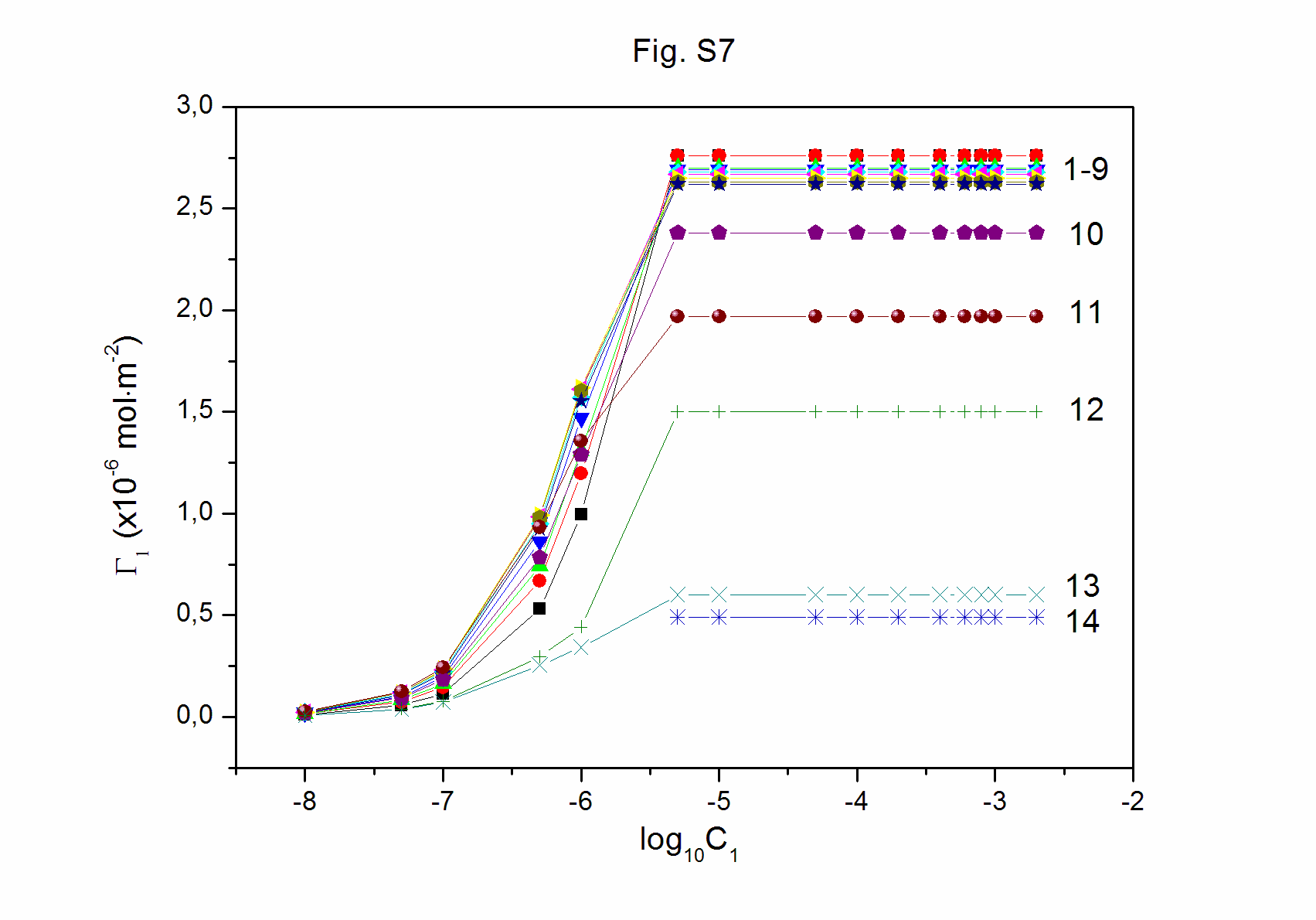


**Fig. S7.** A plot of the Gibbs surface excess concentration of TX-100 at the water-air interface ($\Gamma_{1}$) calculated from Eq. (7) at constant RL concentration vs. logarithm_10_ of TX-100 concentration in the bulk phase ($C_{1}$). Curves 1 - 14 correspond to the constant RL concentration equal to 0.0002, 0.0005, 0.00125, 0.003, 0.00625, 0.01, 0.02, 0.05, 0.125, 0.5, 1, 5, 10 and 20 mgdm^-3^.

**
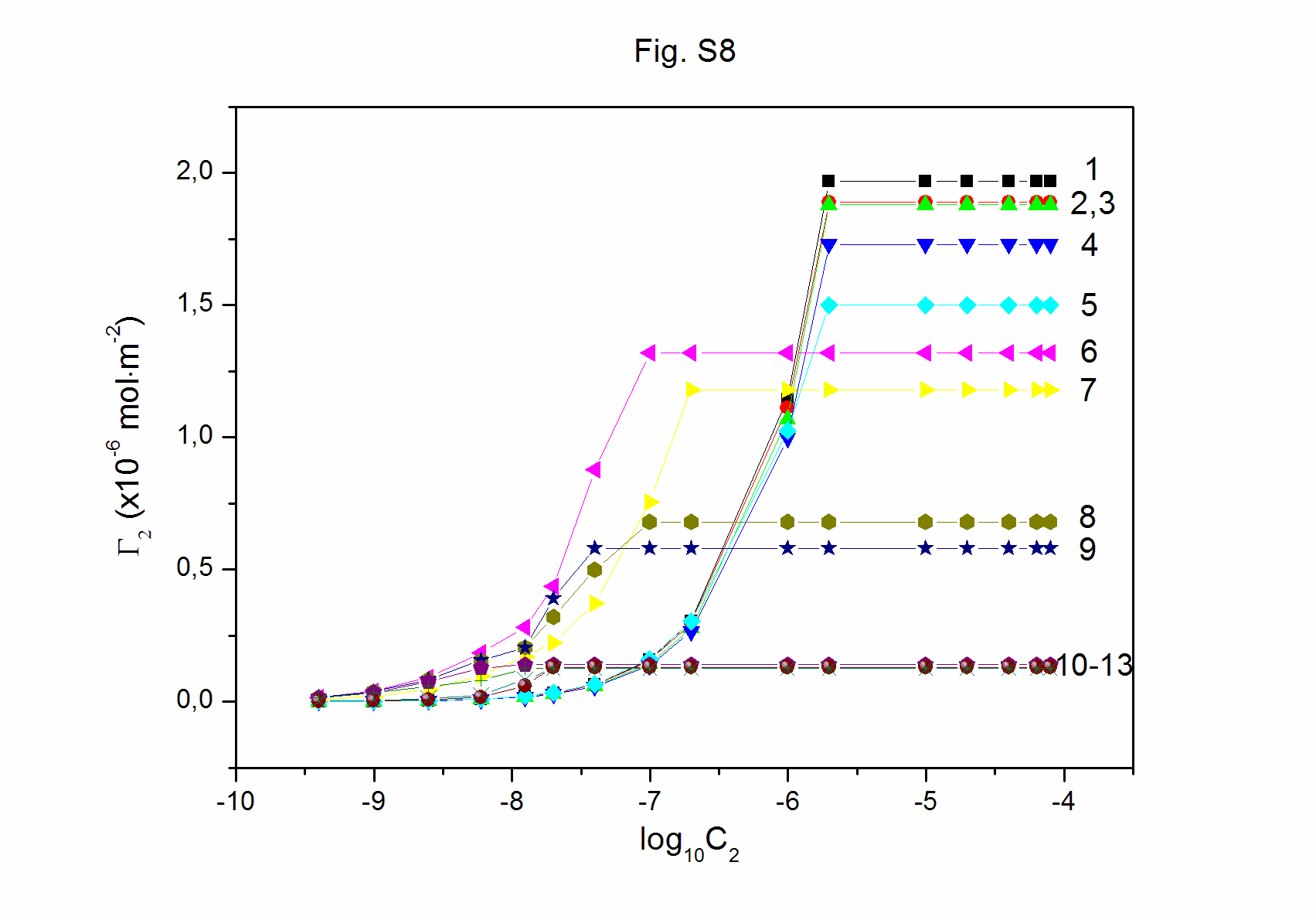
**

**Fig. S8.** A plot of the Gibbs surface excess concentration of RL at the water-air interface ($\Gamma_{2}$) calculated from Eq. (7) at constant TX-100 concentration vs. logarithm_10_ of RL concentration in the bulk phase ($C_{2}$). Curves 1 - 13 correspond to the constant
TX-100 concentration equal to 1 x 10^-8^, 5 x 10^-8^, 1 x 10^-7^, 5 x 10^-7^, 1 x 10^-6^, 5 x 10^-6^,
1 x 10^-5^, 5 x 10^-5^, 1 x 10^-4^, 2 x 10^-4^, 4 x 10^-4^, 6 x 10^-4^, 8 x 10^-4^ moldm^-3^.

**
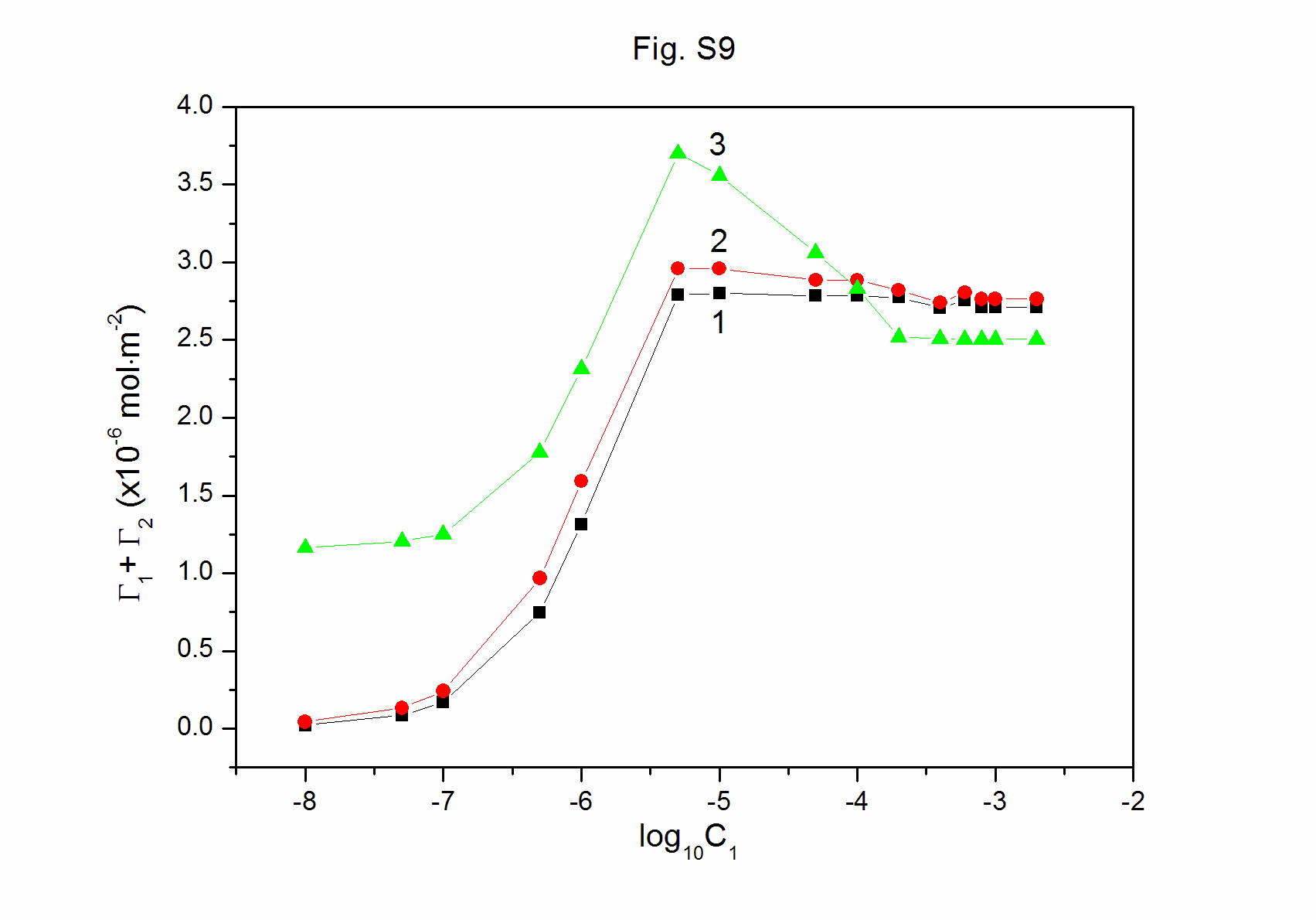
**

**Fig. S9.** A plot of the sum of TX-100 and RL Gibbs surface excess concentration at the
water-air interface ($\Gamma_{1}$+$\Gamma_{2}$) at the constant RL concentration vs. the logarithm_10_ of
TX-100 concentration in the bulk phase ($C_{1}$). Curves 1 - 3 correspond to the constant RL concentration equal to 0.00125 (2.48 x 10^-9^ moldm^-3^), 0.00625 (1.24 x 10^-8^ moldm^-3^) and 0.5 mgdm^-3^ (9.92 x 10^-7^ moldm^-3^), respectively.

**
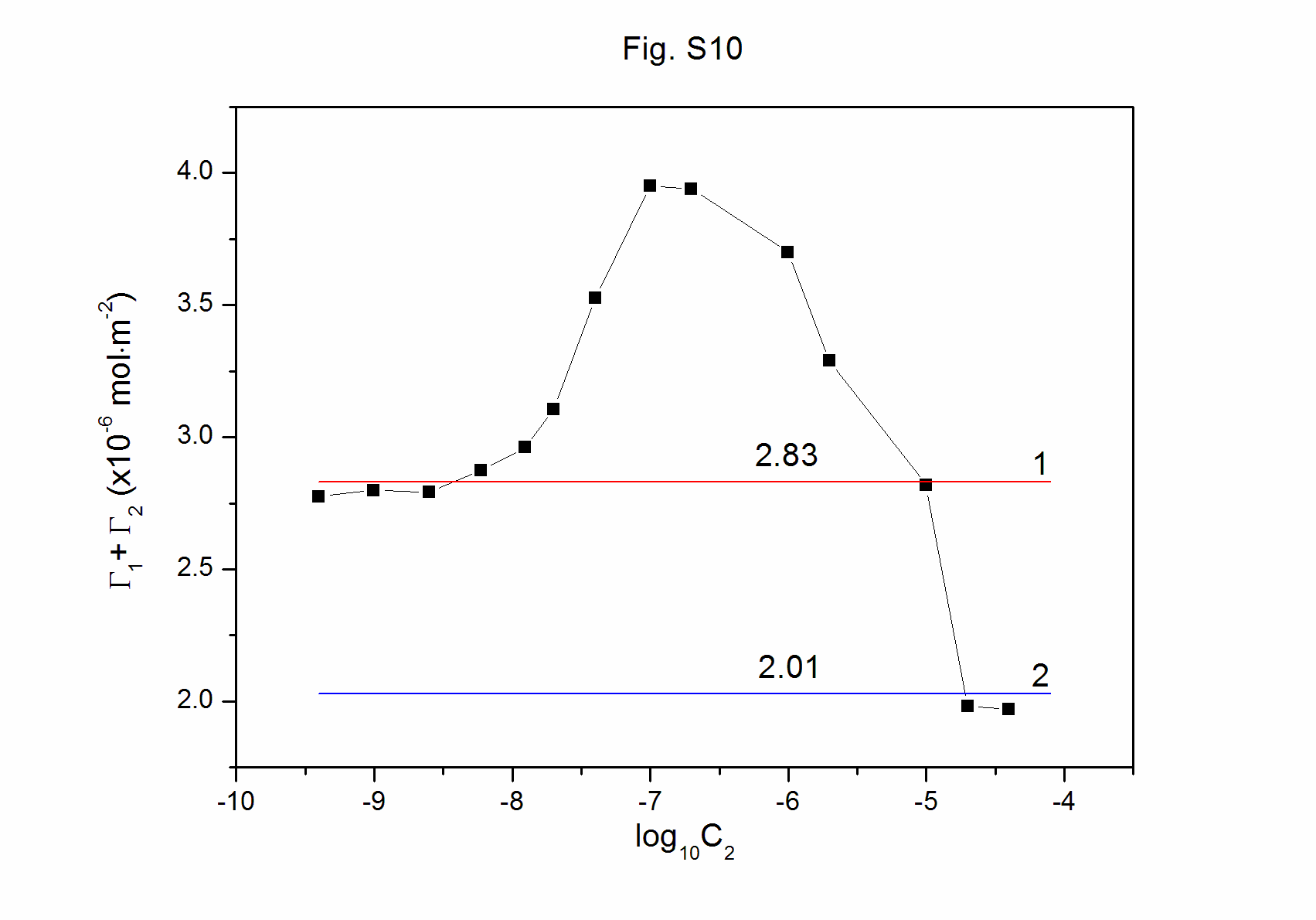
**

**Fig. S10.** A plot of the sum of TX-100 and RL Gibbs surface excess concentration at the water-air interface ($\Gamma_{1}$+$\Gamma_{2}$) vs. the logarithm_10_ of RL concentration in the bulk phase ($C_{2}$). The particular points of this plot correspond to the maximal values from the dependence between the sum of RL and TX-100 Gibbs surface excess concentration at a given constant value of RL concentration. Straight lines 1 and 2 correspond to the maximal value of the Gibbs surface excess concentration of the individual TX-100 and RL, respectively.


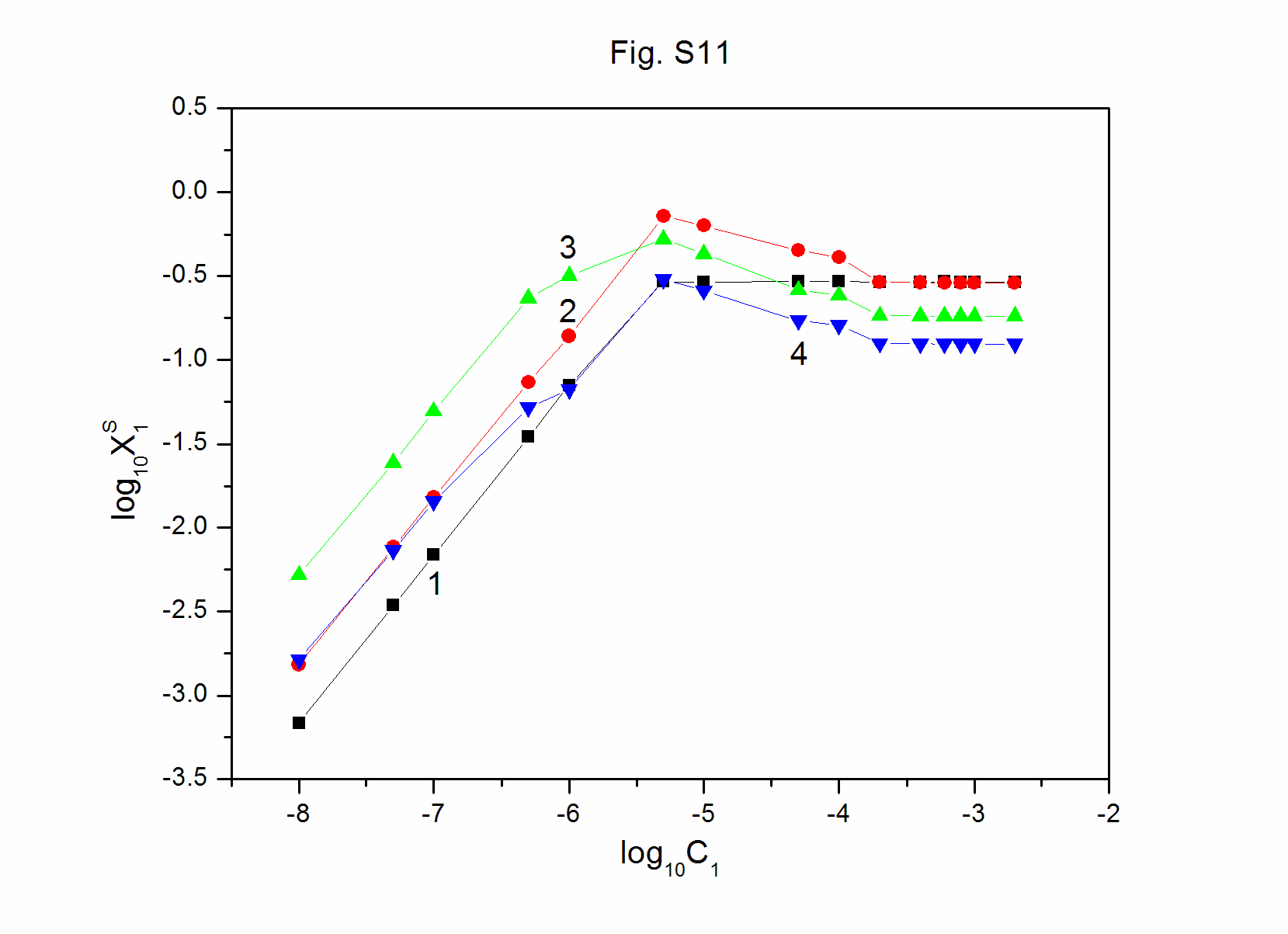


**Fig. S11.** The dependence between the logarithm_10_ of TX-100 mole fraction area occupied by TX-100 in the mixed monolayer ($X_{1}^{S}$) calculated from Eq. (S11) at the constant RL concentration and the logarithm_10_ of TX-100 concentration in the bulk phase ($C_{1}$). Curves 1 - 4 correspond to the constant RL concentration equal to 0.0002
(3.97 x 10^-10^ moldm^-3^), 0.05 (9.92 x 10^-8^ moldm^-3^), 1 (1.98 x 10^-6^ moldm^-3^) and 5 mgdm^-3^ (9.92 x 10^-6^ moldm^-3^), respectively.

**
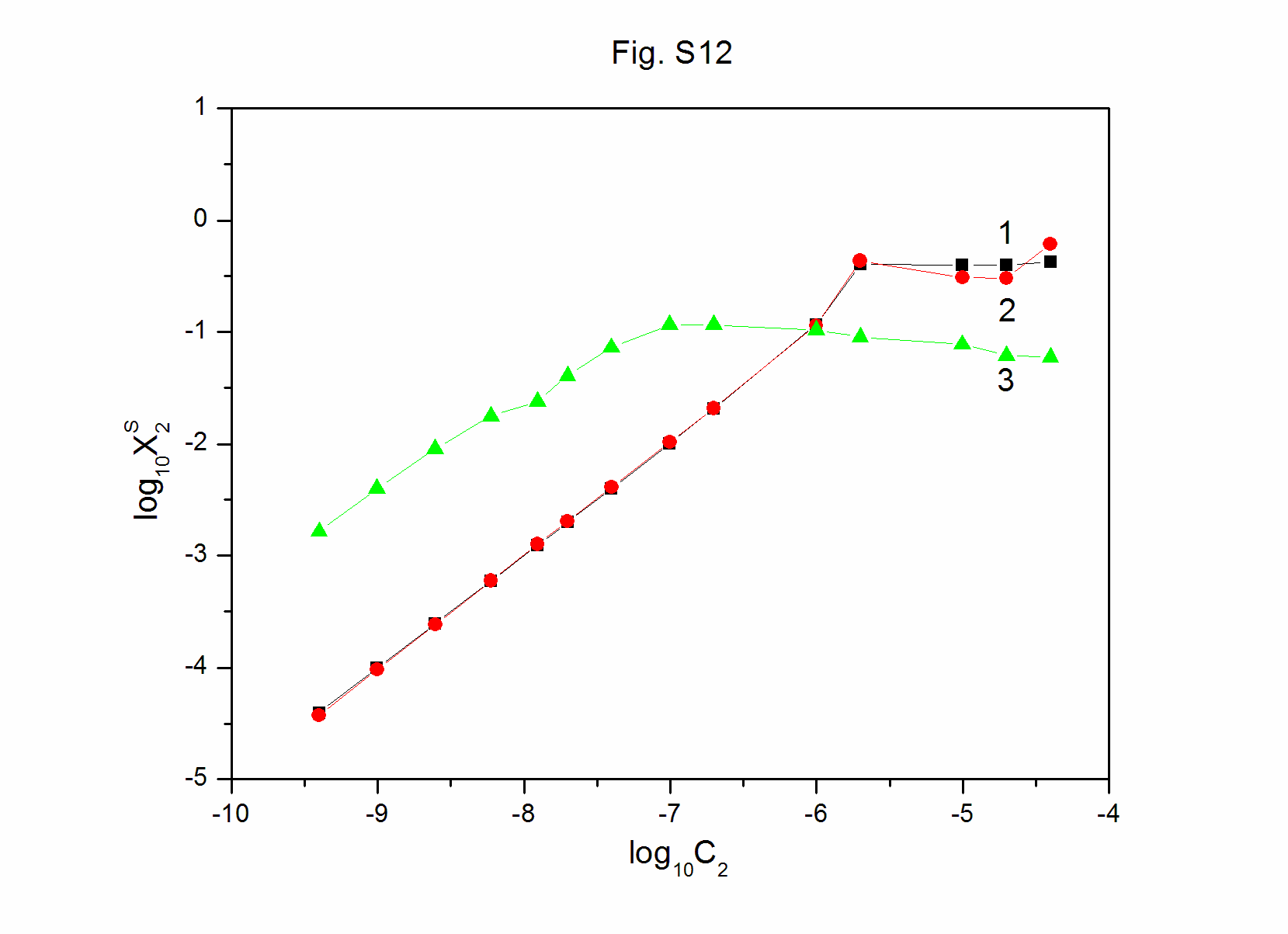
**

**Fig. S12.** The dependence between the logarithm_10_ of RL mole fraction area occupied by RL in the mixed monolayer ($X_{2}^{S}$) calculated from Eq. (S12) at the constant TX-100 concentration and the logarithm_10_ of RL concentration in the bulk phase ($C_{2}$). Curves
1 - 3 correspond to the constant TX-100 concentration equal to 1 x 10^-8^, 5 x 10^-7^ and
5 x 10^-5^ moldm^-3^, respectively.

**
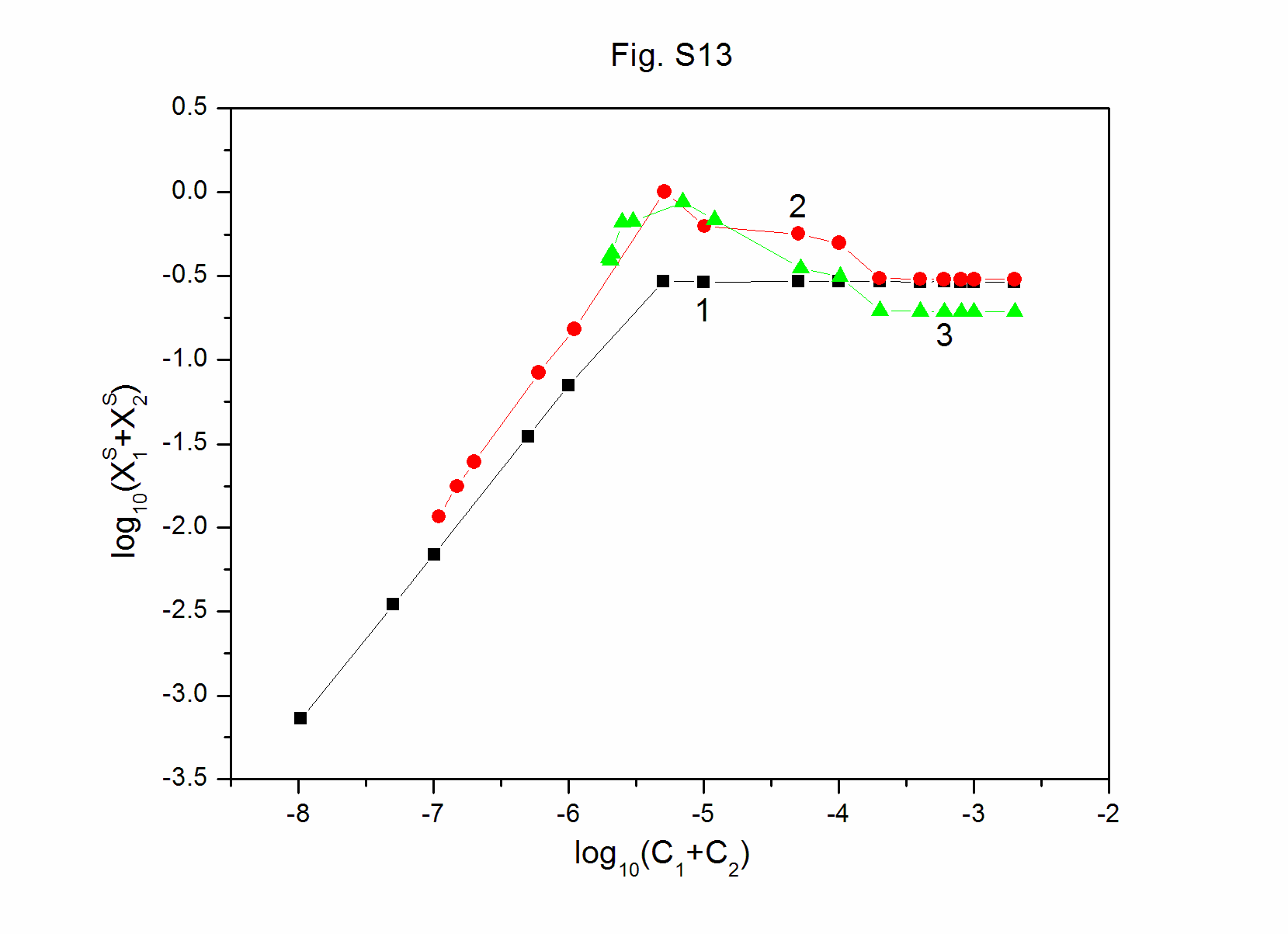
**

**Fig. S13.** The dependence between the logarithm of the sum of mole fraction area occupied by TX-100 and RL in the mixed monolayer ($X_{1}^{S}$+$X_{2}^{S}$) at the constant RL concentration and the sum of the logarithm_10_ of TX-100 and RL concentration in the bulk phase ($C_{1}$+$C_{2}$). Curves 1 - 3 correspond to the constant RL concentration equal to 0.0002 (3.97 x 10^-10^ moldm^-3^), 0.05 (9.92 x 10^-8^ moldm^-3^) and 1 mgdm^-3^ (1.98 x 10^-6^ moldm^-3^), respectively.

**
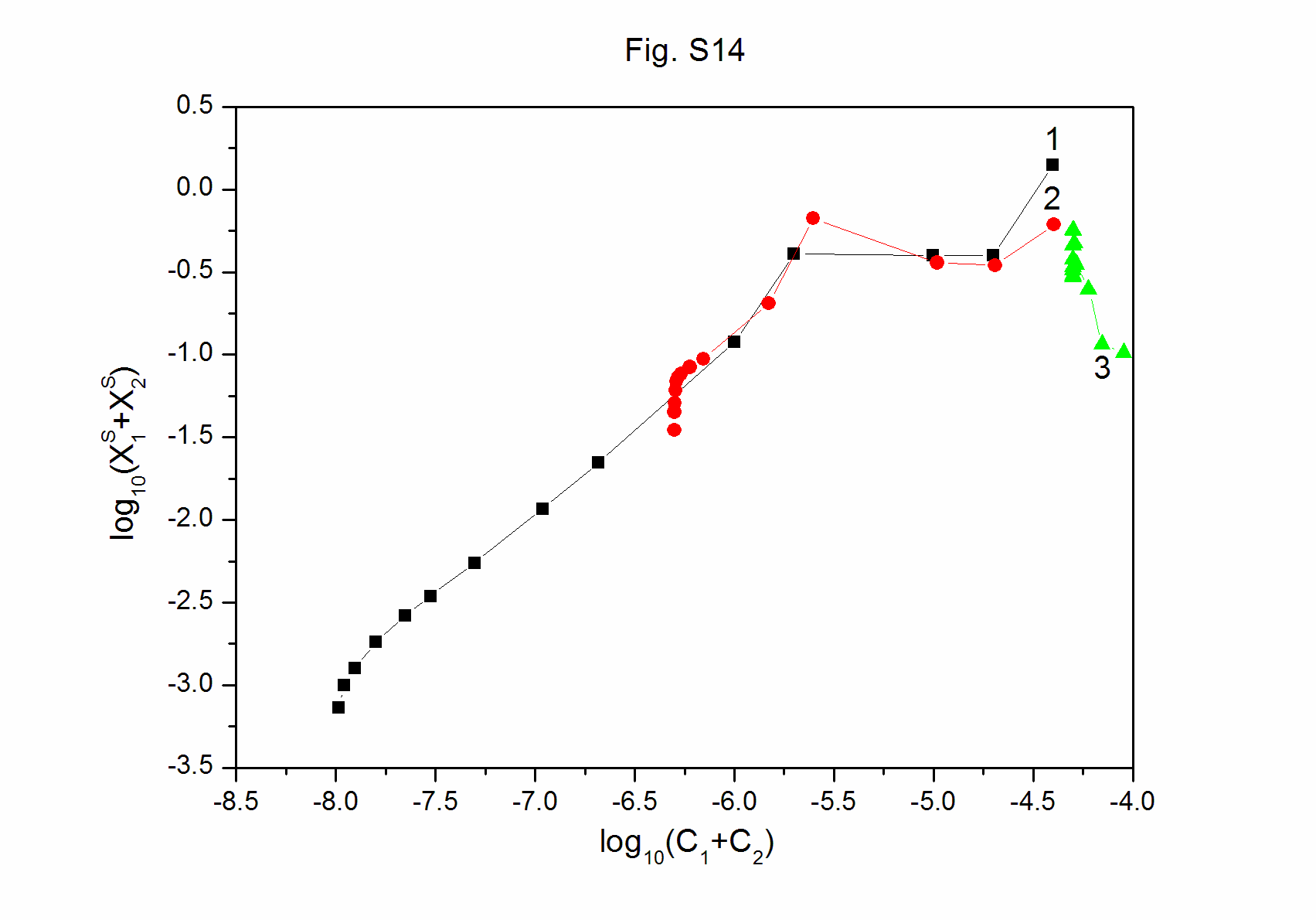
**

**Fig. S14.** The dependence between the logarithm of the sum of mole fraction area occupied by TX-100 and RL in the mixed monolayer ($X_{1}^{S}$+$X_{2}^{S}$) at the constant TX-100 concentration and the sum of the logarithm_10_ of TX-100 and RL concentration in the bulk phase ($C_{1}$+$C_{2}$). Curves 1 - 3 correspond to the constant TX-100 concentration equal to 1 x 10^-8^, 5 x 10^-7^, 5 x 10^-5^ moldm^-3^, respectively.


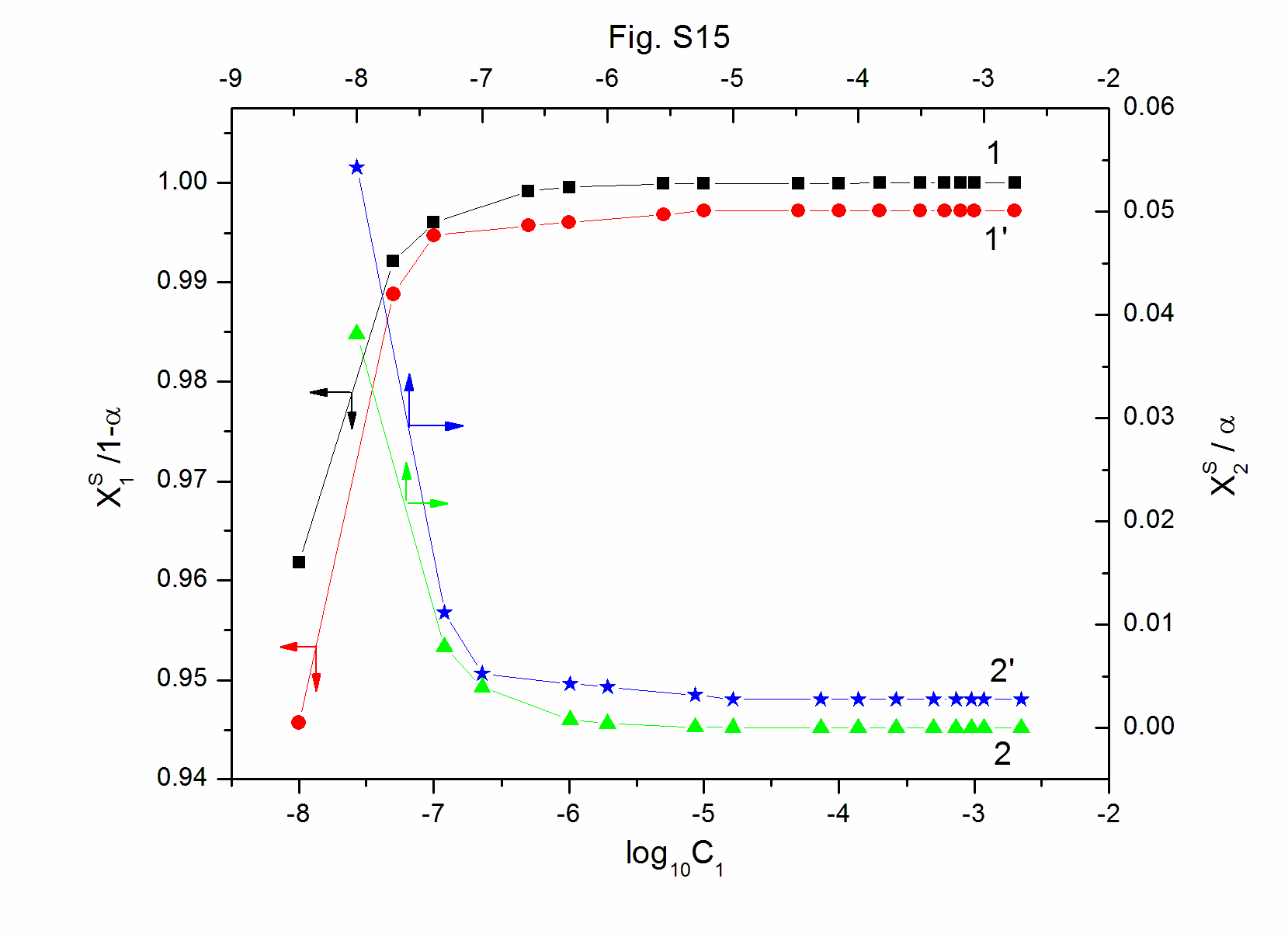


**Fig. S15.** A plot of the mole fraction of TX-100 (curves 1 and 1’) and RL (curves 2 and 2’) in their mixture in the monolayer at the water-air interface ($X_{1}^{S}$ or $X_{2}^{S}$ ) (curves 1 and 2) and in the bulk phase ($1-\alpha$ or $\alpha$) (curves 1’ and 2’) at the constant RL concentration equal to 0.0002 mgdm^-3^ (3.97 x 10^-10^ moldm^-3^) vs. the logarithm of TX-100 concentration in the bulk phase ($C_{1}$).

**
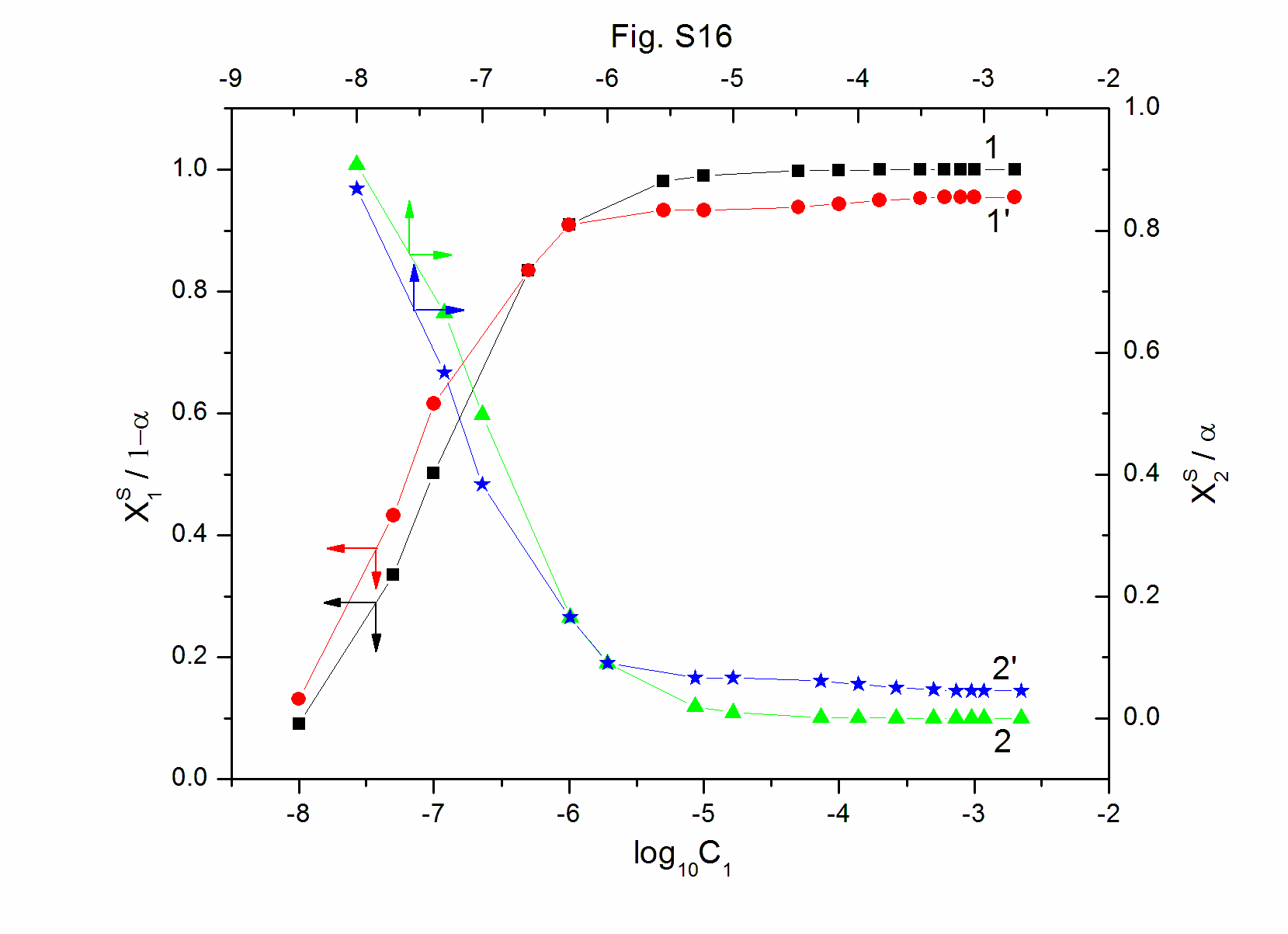
**

**Fig. S16.** A plot of the mole fraction of TX-100 (curves 1 and 1’) and RL (curves 2 and 2’) in their mixture in the monolayer at the water-air interface ($X_{1}^{S}$ or $X_{2}^{S}$ ) (curves 1 and 2) and in the bulk phase ($1-\alpha$ or $\alpha$) (curves 1’ and 2’) at the constant RL concentration equal to 0.05 mgdm^-3^ (9.92 x 10^-8^ moldm^-3^) vs. the logarithm_10_ of TX-100 concentration in the bulk phase ($C_{1}$).

**
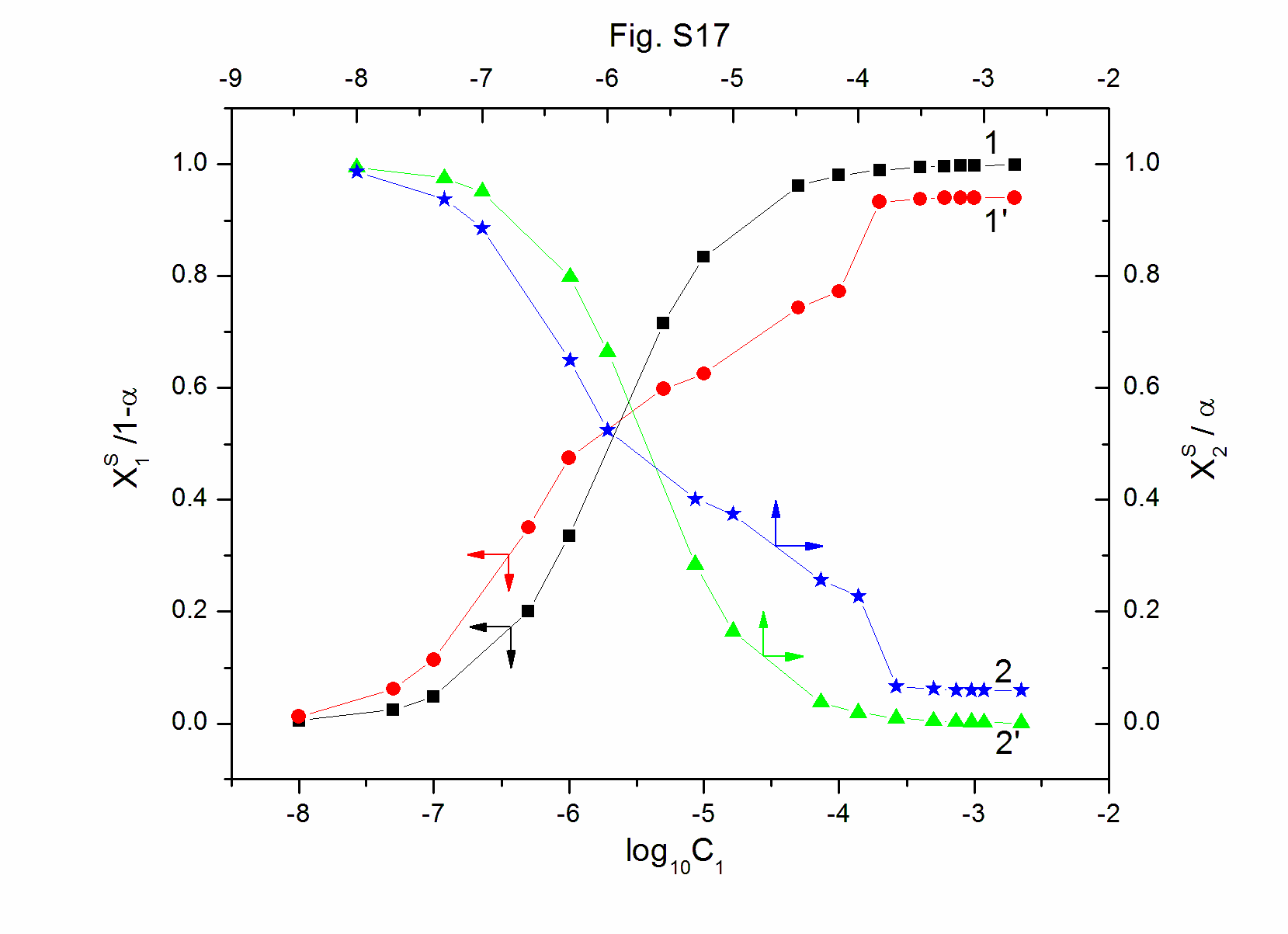
**

**Fig. S17.** A plot of the mole fraction of TX-100 (curves 1 and 2) and RL (curves 2 and 2’) in their mixture in the monolayer at the water-air interface ($X_{1}^{S}$ or $X_{2}^{S}$ ) (curves 1 and 2) and the bulk phase ($1-\alpha$ or $\alpha$) (curves 1’ and 2’) at the constant RL concentration equal to 1 mgdm^-3^ (1.98 x 10^-6^ moldm^-3^) vs. the logarithm_10_ of TX-100 concentration in the bulk phase ($C_{1}$).

**
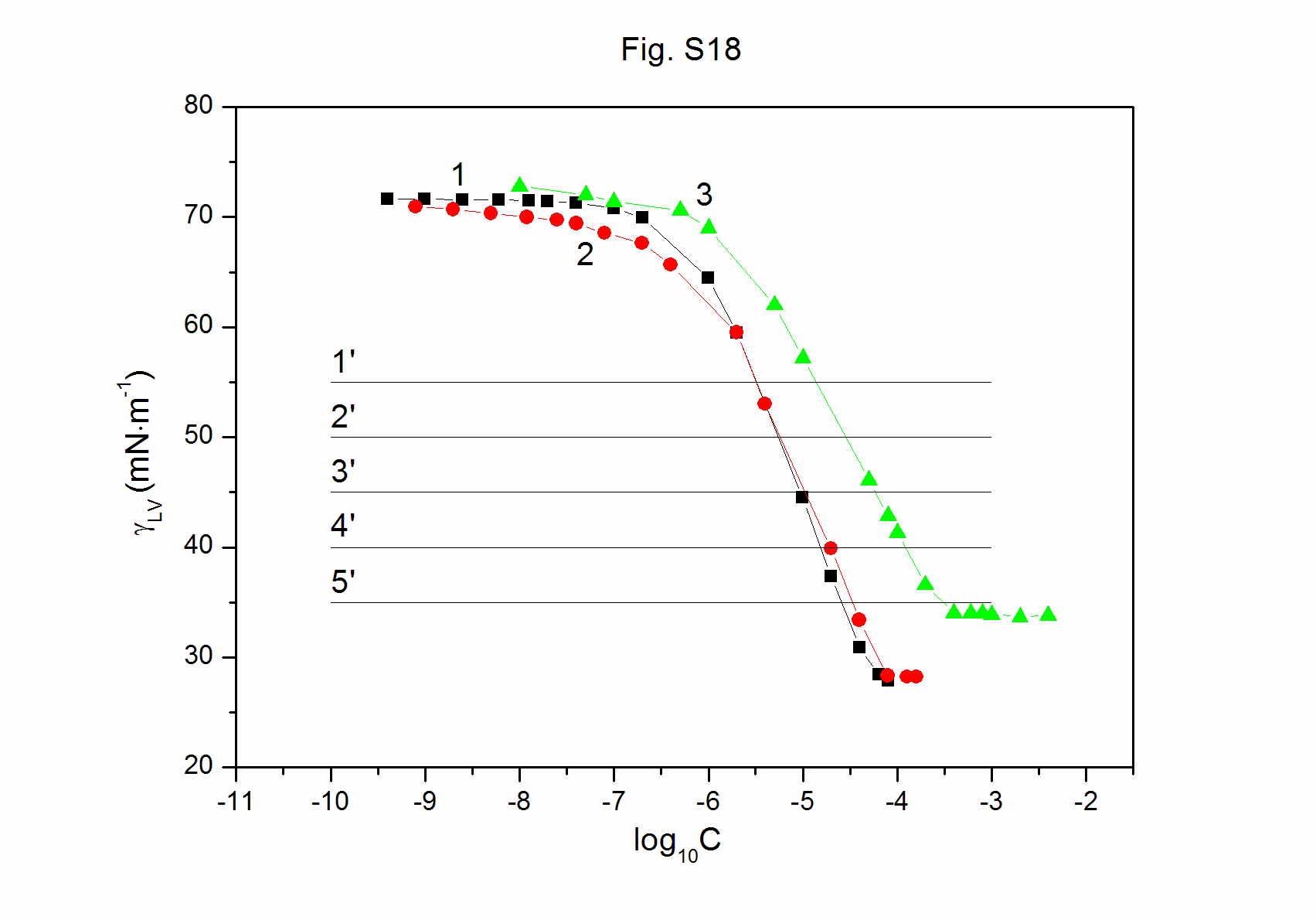
**

**Fig. S18.** A plot of the surface tension ($\gamma_{LV}$) of aqueous solution of RL (curve 1), TX-100 (curve 3) and their mixture (curve 2) for RL mole fraction in the bulk phase equal to 0.5 vs. the logarithm_10_ of RL, TX-100 or mixture concentration ($C$). The straight lines 1’ - 5’ correspond to the given values of solution surface tension (55, 50, 45, 40 and
35 mNm^-1^, respectively).


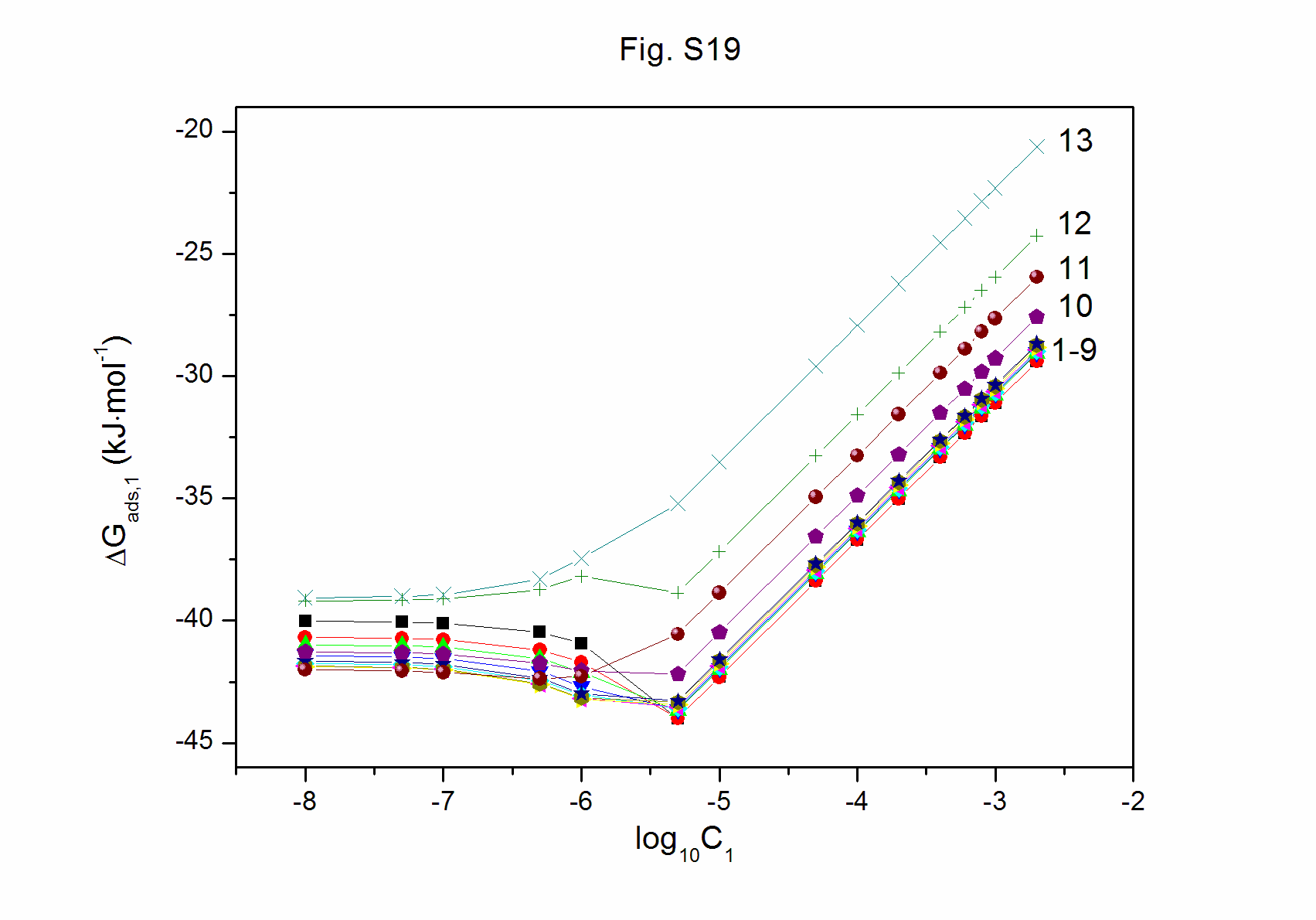


**Fig. S19.** A plot of the standard Gibbs free energy of adsorption of TX-100 ($\Delta G_{ads,1}^{o}$) at the constant RL concentration vs. logarithm_10_ of TX-100 concentration in the bulk phase ($C_{1}$). Curves 1 - 13 correspond to RL concentration equal to 0.0002, 0.0005, 0.00125, 0.003, 0.00625, 0.01, 0.02, 0.05, 0.125, 0.5, 1, 5 and 10 mgdm^-3^.

**
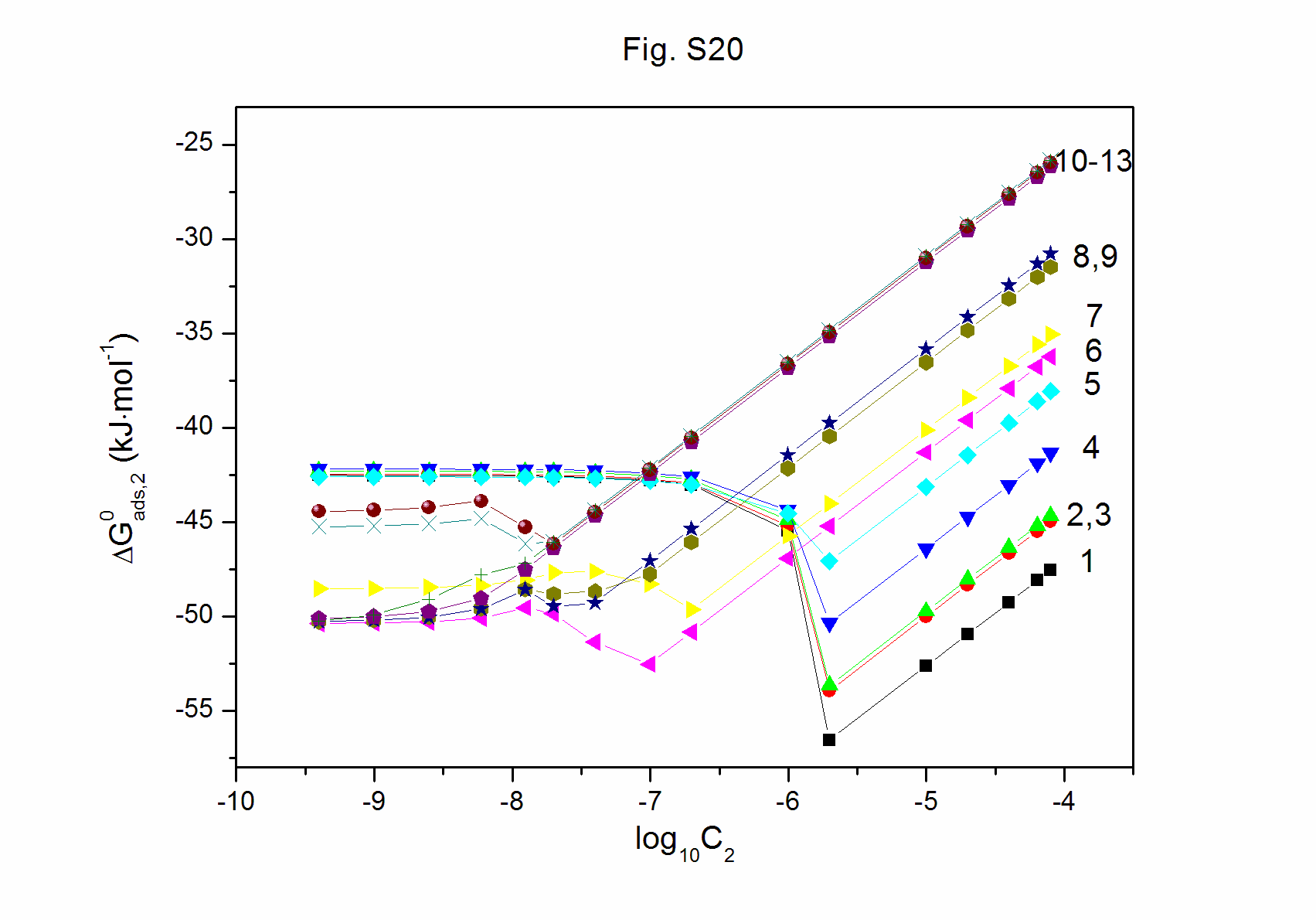
**

**Fig. S20.** A plot of the standard Gibbs free energy of adsorption of RL ($\Delta G_{ads,2}^{o}$) at the constant TX-100 concentration vs. the logarithm_10_ of RL concentration in the bulk phase ($C_{2}$). Curves 1 - 13 correspond to the TX-100 concentration equal to 1 x 10^-8^, 5 x 10^-8^, 1 x 10^-7^, 5 x 10^-7^, 1 x 10^-6^, 5 x 10^-6^, 1 x 10^-5^, 5 x 10^-5^, 1 x 10^-4^, 2 x 10^-4^, 4 x 10^-4^,
6 x 10^-4^, 8 x 10^-4^ moldm^-3^.

**
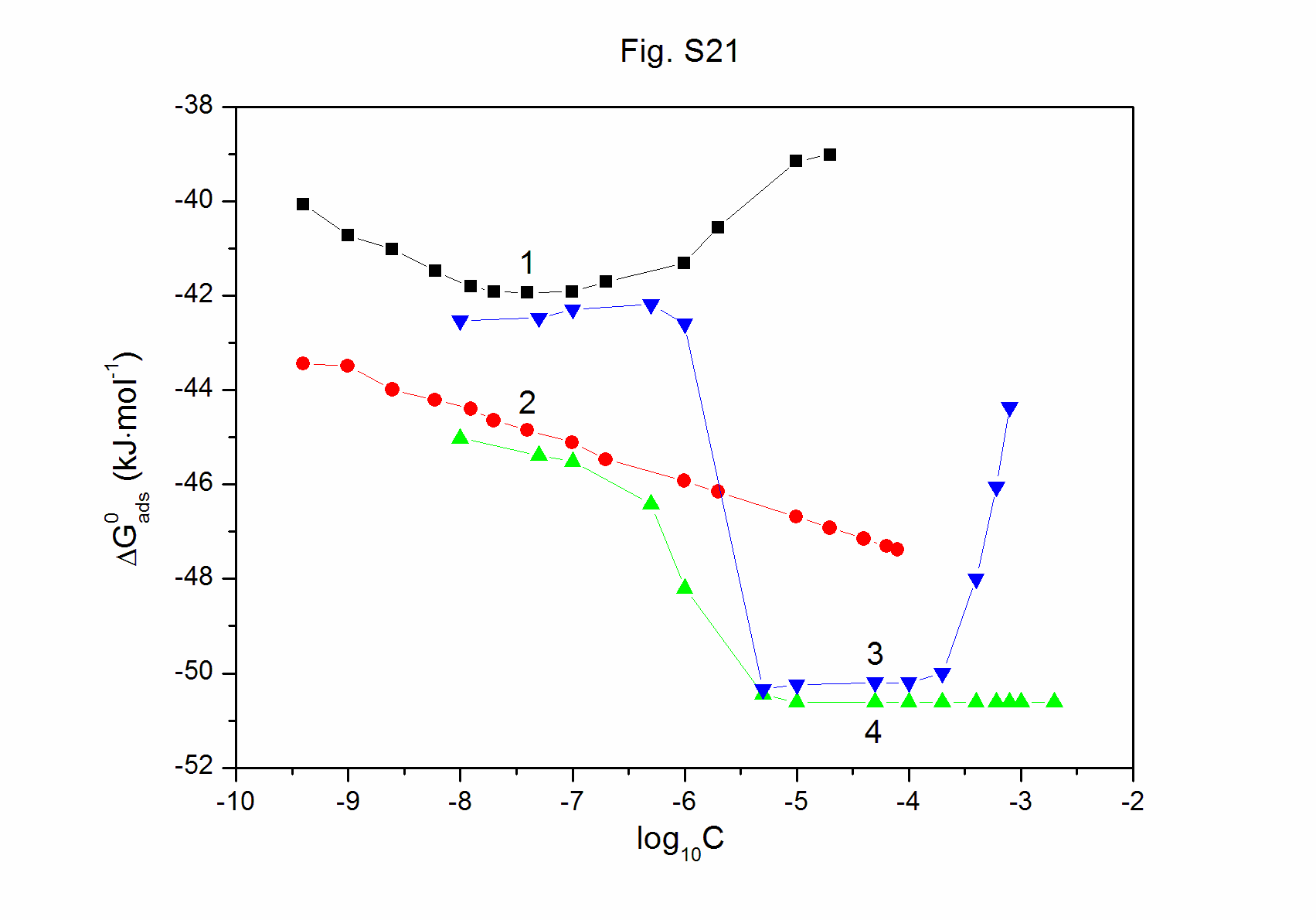
**

**Fig. S21.** The dependence between the standard Gibbs free energy of adsorption ($\Delta G_{ads}^{o}$) of TX-100 (curves 1 and 2) and RL (curves 3 and 4) calculated from Eqs. (15) and (16), respectively and the logarithm_10_ of TX-100 or RL concentration in the bulk phase ($C$). The points on the curves 1 and 3 correspond to the constant values of $\Delta G_{ads}^{o}$ at the low surfactant concentration (see Figs. S19 and S20, respectively).

**
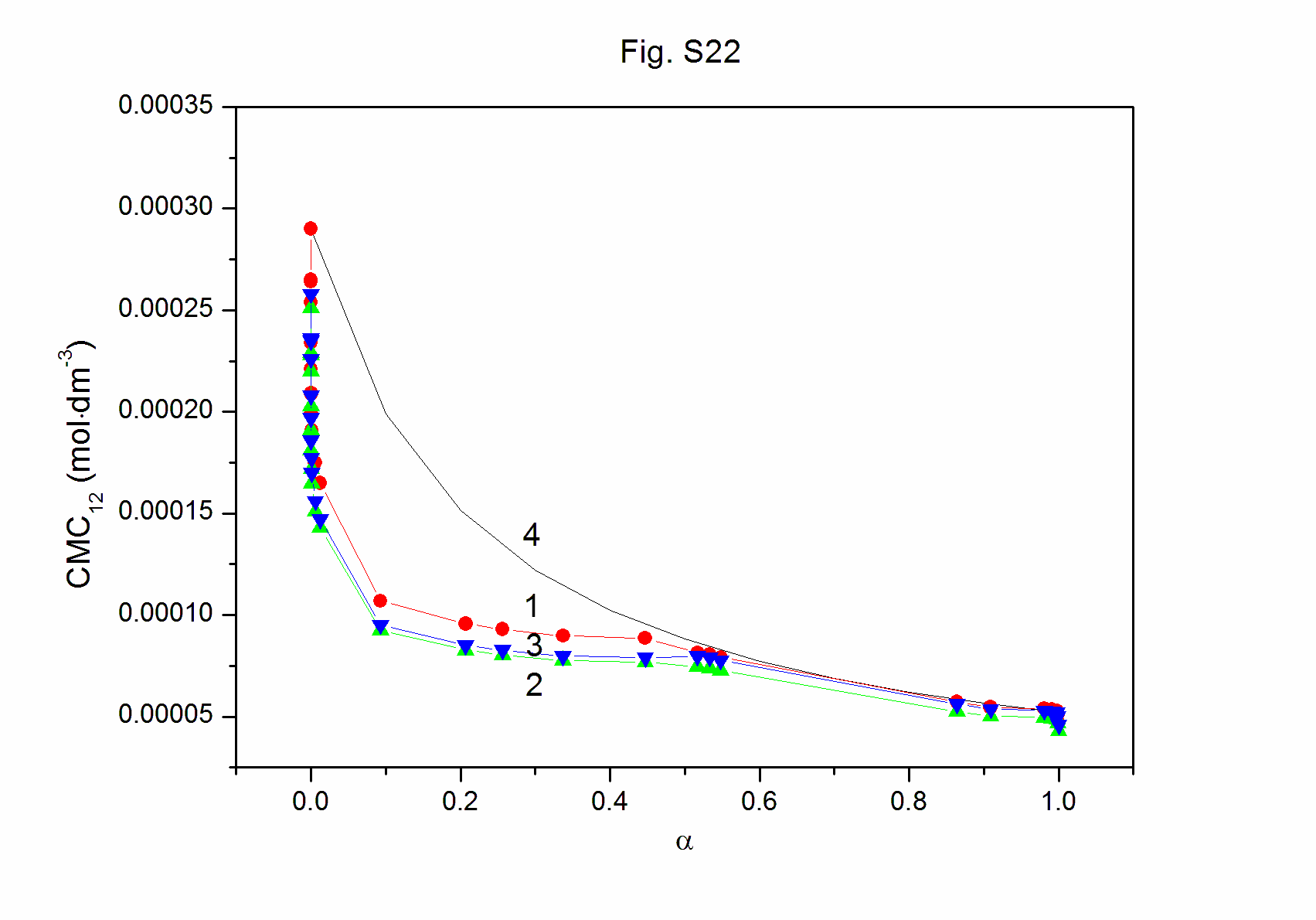
**

**Fig. S22.** A plot of the critical micelle concentration of aqueous solution of TX-100 and RL mixture ($CMC_{12}$) determined on the basis of surface tension (curve 1), density (curve 2), viscosity (curve 3) isotherms and calculated from Eq. (17) (curve 4) vs. RL mole fraction in surfactant mixture in the bulk phase ($\alpha$).

**
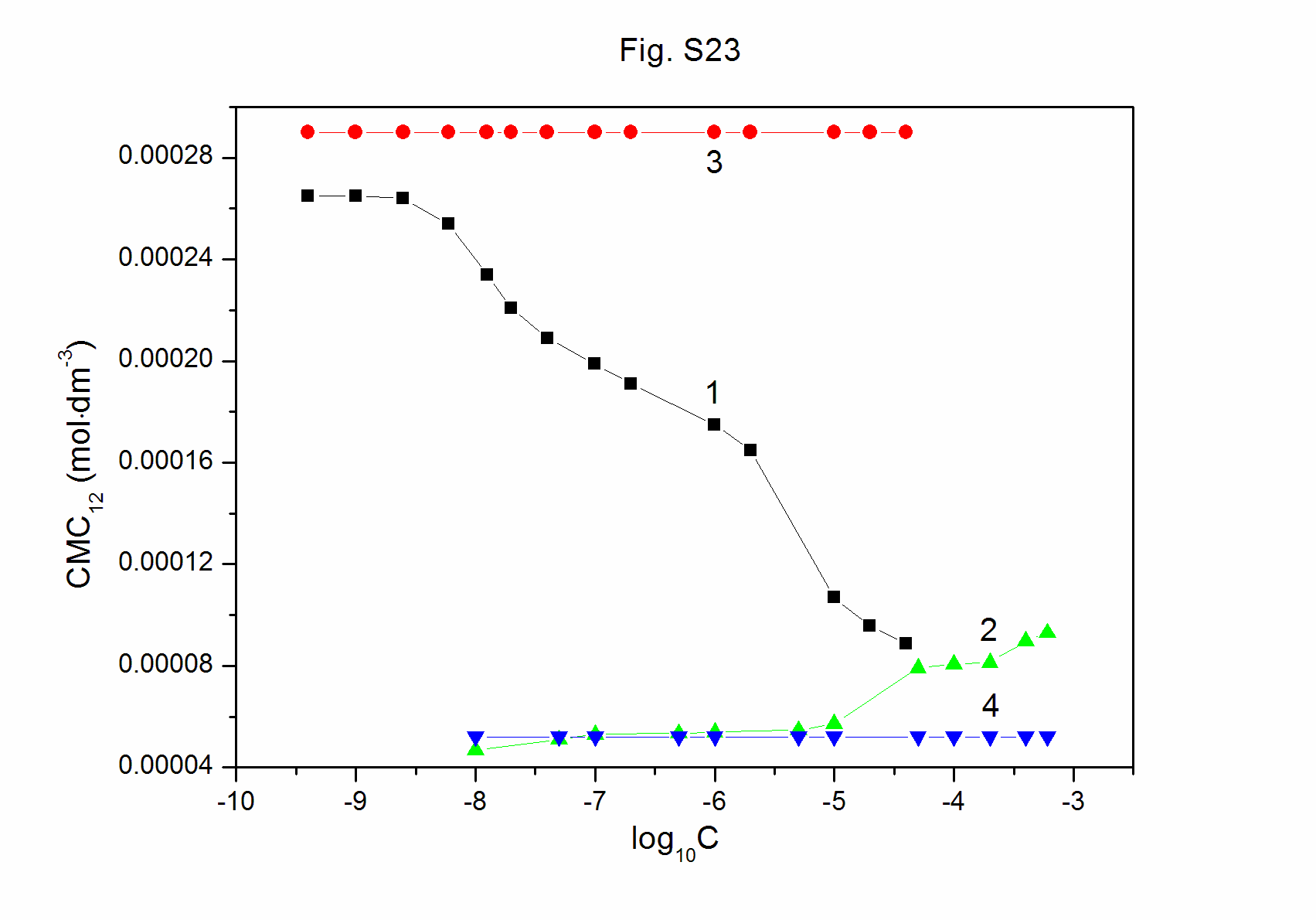
**

**Fig. S23.** A plot of the critical micelle concentration (CMC) of aqueous solution of TX-100 and RL mixture ($CMC_{12}$) vs. the logarithm_10_ of RL (curve 1) or TX-100 (curve 2) concentration in the bulk phase ($C$). Curves 3 and 4 correspond the value of CMC of individual TX-100 and RL, respectively.

**
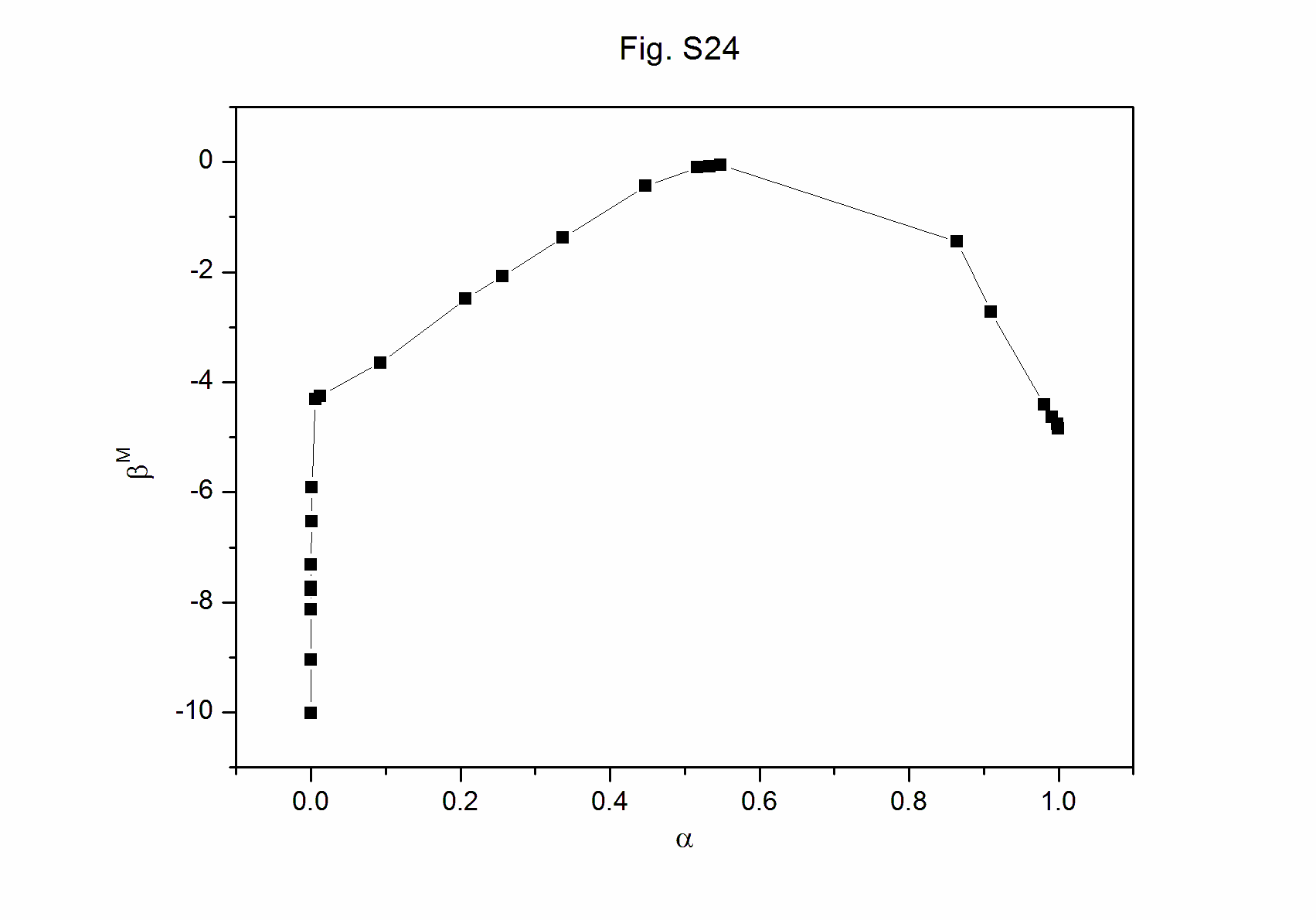
**

**Fig. S24.** The dependence between the parameter of intermolecular interactions in the mixed micelle of TX-100 and RL ($\beta^{M}$) and the mole fraction of RL ($\alpha$) in surfactant mixture in the bulk phase.

**
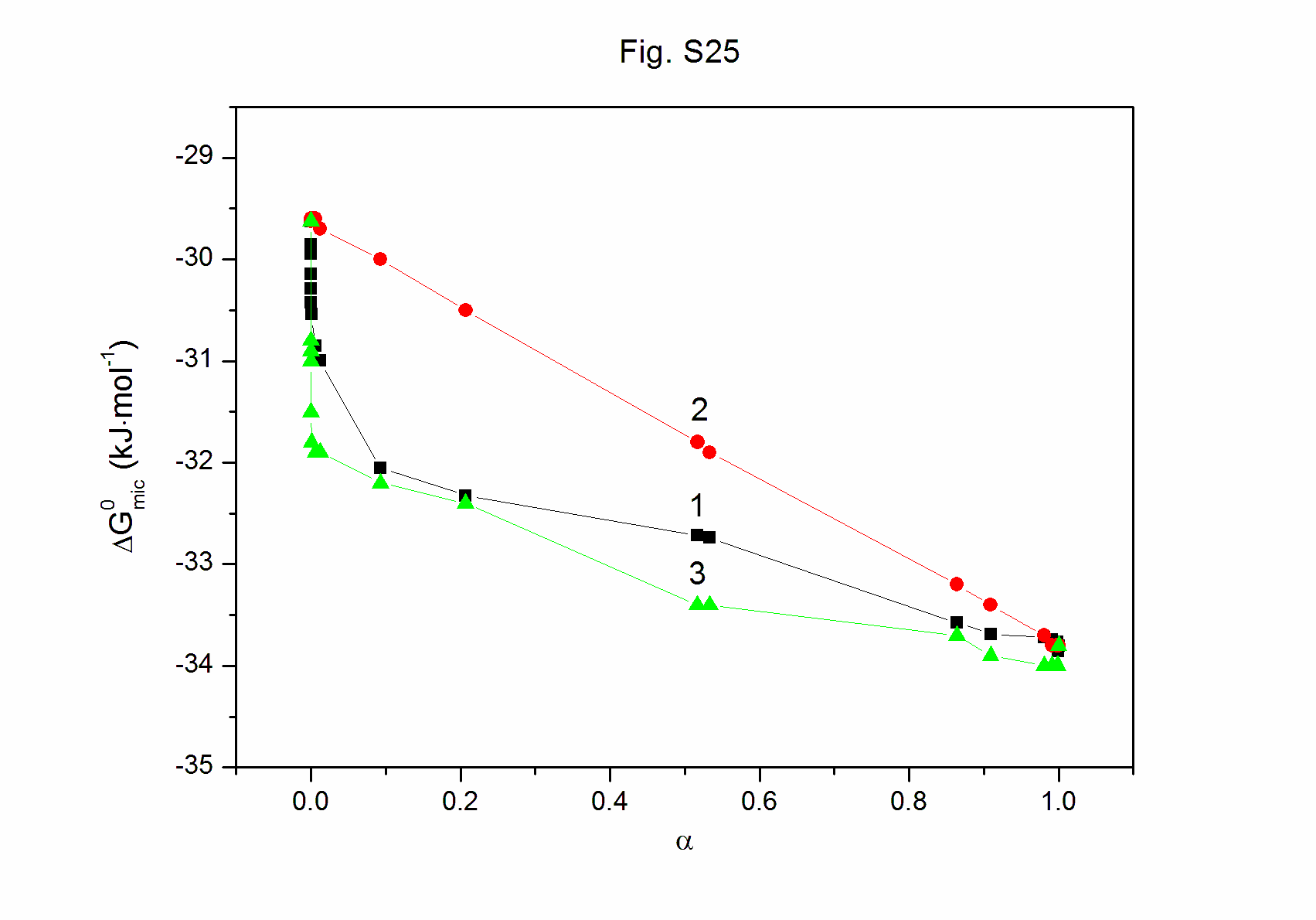
**

**Fig. S25.** A plot of the standard Gibbs free energy of surfactants mixture micellization ($\Delta G_{mic}^{o}$) vs. the mole fraction of RL ($\alpha$) in surfactant mixture in the bulk phase. Curve 1 corresponds to the values of $\Delta G_{mic}^{o}$ obtained on the basis of CMC determined from the surface tension isotherms, curve 2 to the values calculated for ideal surfactant mixing and curve 3 to the sum of the $\Delta G_{mic}^{o}$ of ideal mixture and the Gibbs excess free energy of nonideal mixing of surfactants in micelle (Eq. (23)).

**
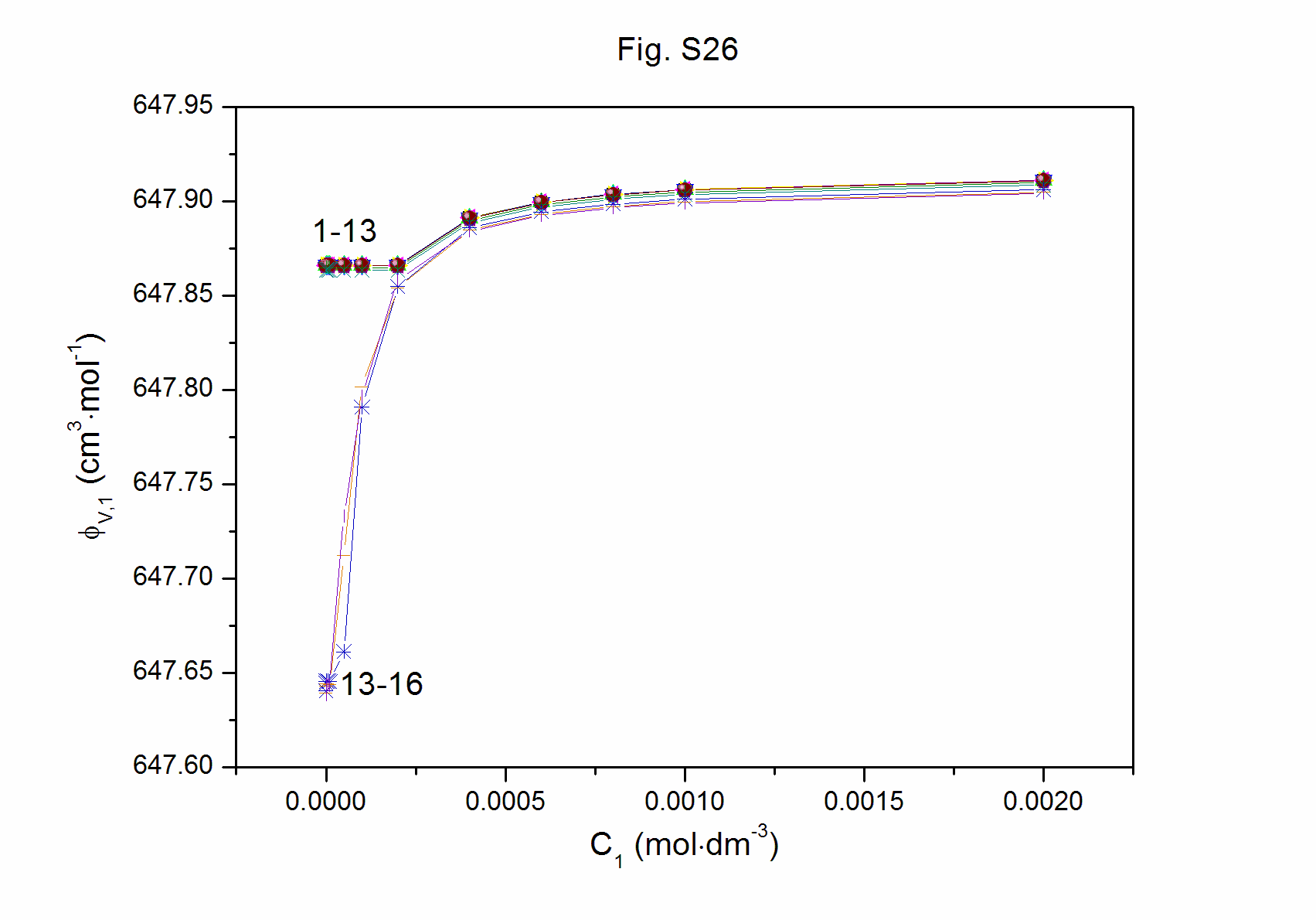
**

**Fig. S26.** A plot of the apparent molar volume of TX-100 ($\varphi_{V,1}$) calculated from Eq. (18) at the constant RL concentration vs. the TX-100 concentration in the bulk phase ($C_{1}$). Curves 1 - 16 correspond to the RL concentrations equal to 0.0002, 0.0005, 0.00125, 0.003, 0.00625, 0.01, 0.02, 0.05, 0.125, 0.5, 1, 5, 10, 20, 32 and 40 mgdm^-3^.

**
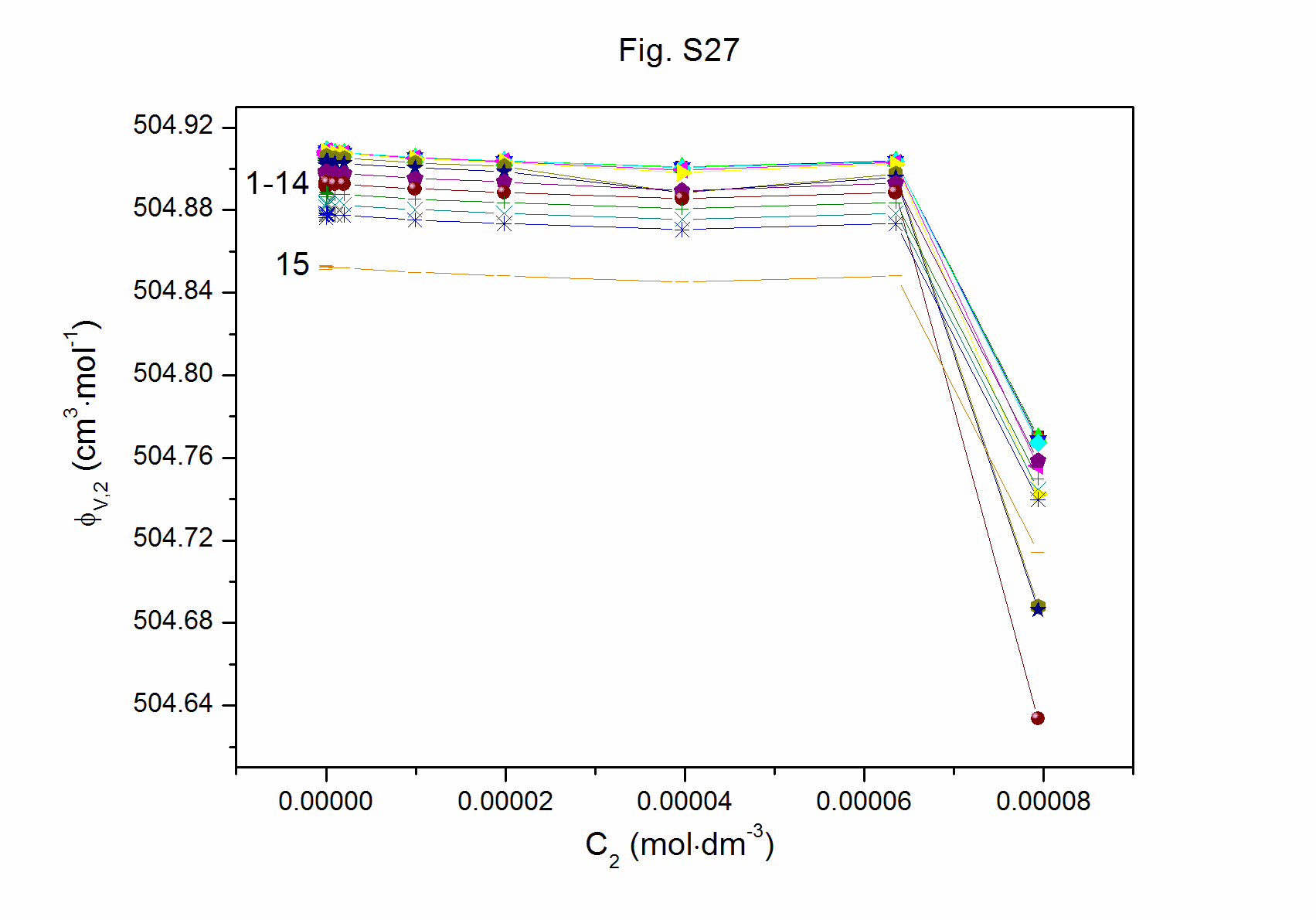
**

**Fig. S27.** A plot of the apparent molar volume of RL ($\varphi_{V,2}$) calculated from Eq. (18) at the constant TX-100 concentration vs. the RL concentration in the bulk phase ($C_{2}$). Curves 1 - 15 correspond to TX-100 concentrations equal to 1 x 10^-8^, 5 x 10^-8^, 1 x 10^-7^, 5 x 10^-7^, 1 x 10^-6^, 5 x 10^-6^, 1 x 10^-5^, 5 x 10^-5^, 1 x 10^-4^, 2 x 10^-4^, 4 x 10^-4^, 6 x 10^-4^,
8 x 10^-4^, 1 x 10^-3^, 2 x 10^-3^ moldm^-3^.

**
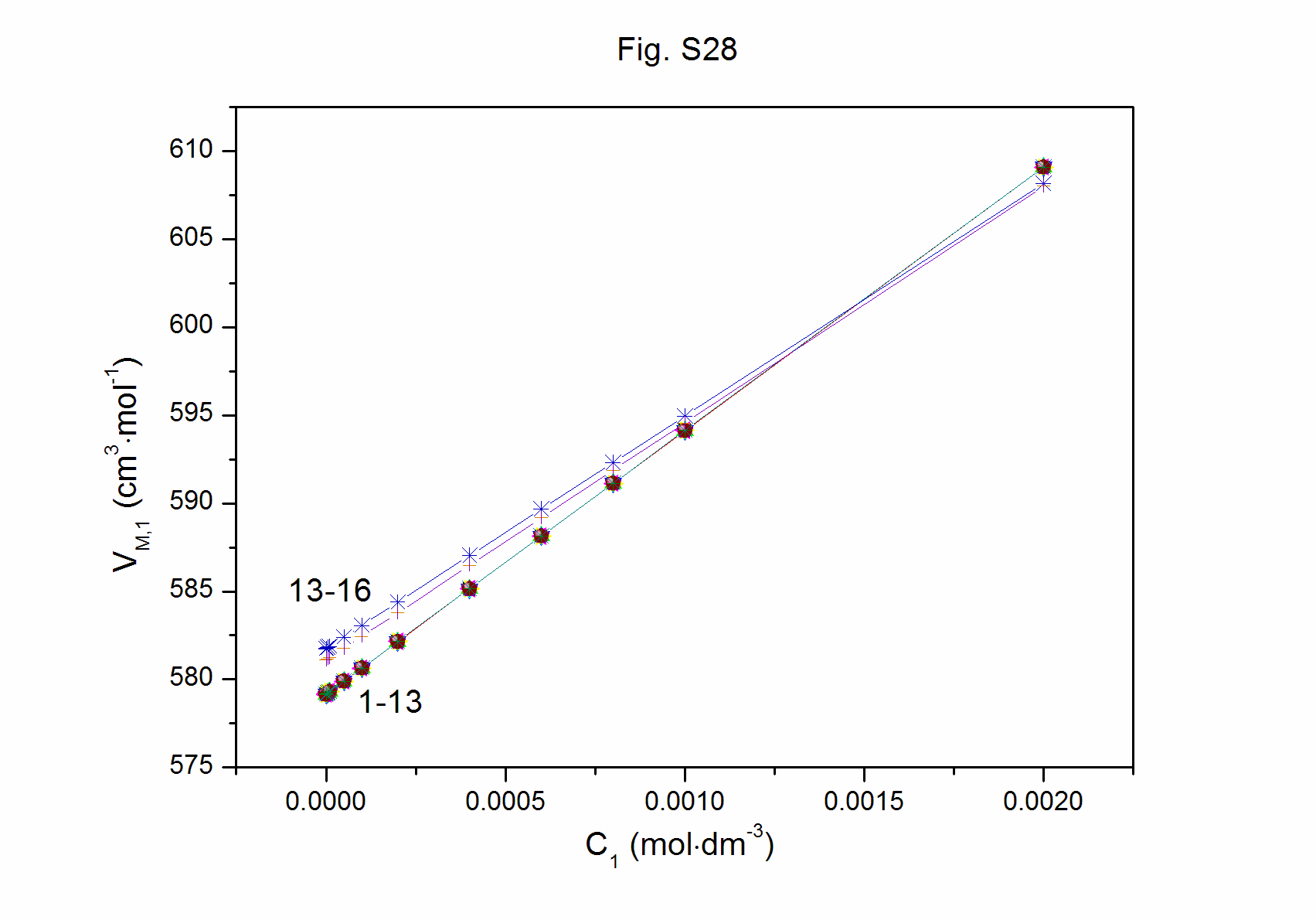
**

**Fig. S28.** A plot of the partial molar volume of TX-100 ($\overline{V}_{M,1}$) calculated from Eq. (19) at the constant RL concentration vs. the TX-100 concentration in the bulk phase ($C_{1}$). Curves 1 - 16 as in Fig. S26.

**
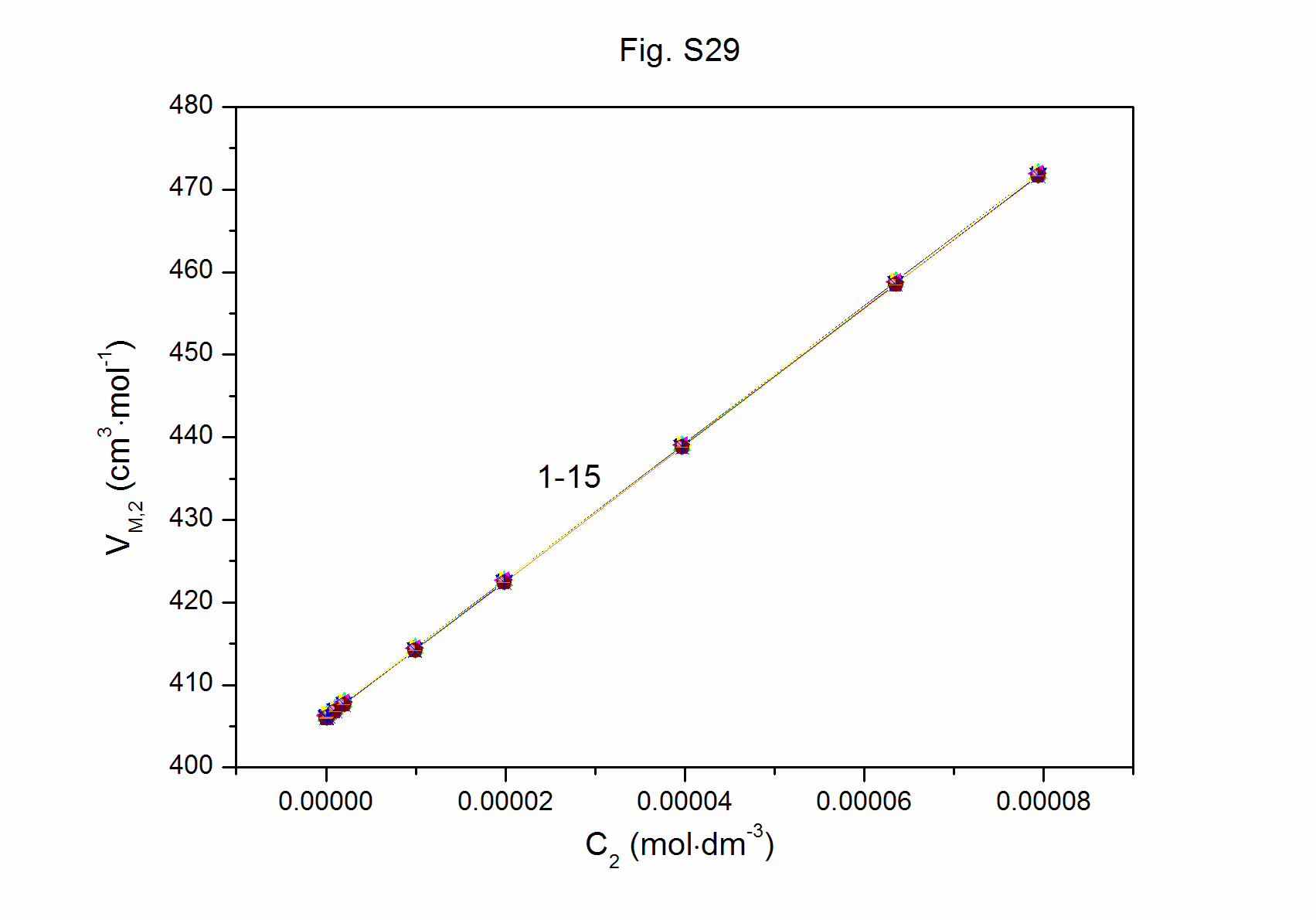
**

**Fig. S29.** A plot of the partial molar volume of RL ($\overline{V}_{M,2}$) calculated from Eq. (19) at the constant TX-100 concentration vs. the RL concentration in the bulk phase ($C_{2}$). Curves 1 - 15 as in Fig. S27.
